# Supplementary material for: What Happened and Why: Responding to Racism, Discrimination, and Microaggressions in the Clinical Learning Environment
Source: MedEdPORTAL. 2022 Nov 1;18:11280. doi: 10.15766/mep_2374-8265.11280 (PMC9622434; doi:10.15766/mep_2374-8265.11280)
Supplement: Supplementary file 1 — Facilitator Guide.docxStudent Guide.docxRDM Faculty Development.pptxGuide for Implementation.docxPreworkshop Survey.docxPostworkshop Survey.docx [file mep_2374-8265.11280-s001.zip › E. Preworkshop Survey.docx]

What Happened and Why:


Responding to Racism, Discrimination, and Microaggressions in the Clinical Learning Environment

TRAINING CURRICULUM: **Workshop Pre-Survey**

Q1

Thank you for participating in this micro-aggression workshop. This session is implemented for medical students as they start their clinical rotations. The goal is to arm students with the tools needed to navigate uncomfortable situations in a clinical setting with mixed power dynamics. Your feedback is necessary to improve this workshop over time and to help us recognize what needs are still unmet in this regard.

In order to collect data anonymously in a way that we can combine data from the pre-and post-session survey as well as future data that will be collected at a later point in time, we ask that each participant create a unique identifying code.

Please list the two digits for your birth month followed by your last two social.
For example, if you were born in January and your social ends in 56 - enter 0156.

After all data is collected and linked your entered ID will be replaced with a unique random study-id so that no link to your unique identifiers will remain.

________________________________________________________________

________________________________________________________________

________________________________________________________________

________________________________________________________________

________________________________________________________________

Q2
The acronym RDM will be used throughout this survey and the workshop. RDM stands for racism, discrimination, and microaggressions. The working definition of microaggression that we will be using during this workshop is: A brief and commonplace daily verbal, behavioral, or environmental indignities, whether intentional or unintentional, that communicate hostile, derogatory, or negative slights and insults toward marginalized groups of people. 

Have you yourself experienced or witnessed an instance of RDM in a clinical setting during your time as a student?

- Yes (1)
- Maybe (2)
- No (3)

Free Response: If you answered yes to the above, please add additional details about your experience.


Q3. How many times have you witnessed or experienced RDM instances directed toward yourself or others?

|  | Never (0) | Once (1) | More than once (2+) |
| --- | --- | --- | --- |
| Yourself (1) |  |  |  |
| Another student or member of the medical team (2) |  |  |  |
| The Patient (3) |  |  |  |

Q4 If you answered yes to any of the above, who was responsible for the micro-aggression in the scenario? Select all that apply.

- Myself (1)
- Attending (2)
- Fellow (3)
- Resident (4)
- Intern (5)
- Medical Student (6)
- Another health professions student (7)
- Another health professional (e.g., nurse, physical therapist, pharmacist) (8)
- Patient (9)
- Other (10) ________________________________________________

Q5 Please indicate your level of agreement with the following statements:

|  | Strongly Disagree (1) | Somewhat disagree (2) | Neither agree nor disagree (3) | Somewhat agree (4) | Strongly agree (5) |
| --- | --- | --- | --- | --- | --- |
| I am aware of different strategies to deal with RDMs aimed at other members of the medical team (1) |  |  |  |  |  |
| I am aware of different strategies to deal with RDMs aimed at myself (2) |  |  |  |  |  |
| I am aware of strategies to deal with RDMs aimed at patients, family members, or other support person(s) (3) |  |  |  |  |  |
| I feel confident in applying communication strategies to deal with RDMs in the clinical learning environment (4) |  |  |  |  |  |

Q6 Please answer each of the following questions based on your current level of comfort for addressing instances of RDMs

|  | Strongly Disagree (1) | Somewhat disagree (2) | Neither agree nor disagree (3) | Somewhat agree (4) | Strongly agree (5) |
| --- | --- | --- | --- | --- | --- |
| I am comfortable addressing RDMs aimed at others (1) |  |  |  |  |  |
| I am comfortable addressing RDMs aimed at myself (2) |  |  |  |  |  |
| I am comfortable addressing RDMs aimed at patients (3) |  |  |  |  |  |
| I am comfortable addressing RDMs I have committed myself (4) |  |  |  |  |  |

| Page Break |  |
| --- | --- |

Q7
I identify as someone who is a member of a demographic that is traditionally under-represented in medicine

- Yes (1)
- No (2)

Optional: If yes, in what way(s)?

________________________________________________________________

________________________________________________________________

Q8 What is your gender identity? (Select any that apply.)

- Female (1)
- Male (2)
- Non-binary (3)
- Trans-man (4)
- Trans-woman (5)
- Gender Non-Conforming (6)
- Other (7) ________________________________________________
- Prefer not to answer (8)

Q9 What is your sexual orientation? (Select any that apply.)

- Asexual (1)
- Bisexual (2)
- Gay (3)
- Heterosexual or straight (4)
- Lesbian (5)
- Pansexual (6)
- Queer (7)
- Other/None of the above (8)
- Prefer not to answer (9)

Q10 What is your racial and ethnic identity? (Select any that apply.)

- Asian (1)
- Black or African American (2)
- Hawaiian Native or other Pacific Islander (3)
- Hispanic or Latina/o/x/e (4)
- Native American or Alaskan Native (5)
- White (6)
- Other (7)
- Prefer not to answer (8)

Q11 What is your current year at medical school?

- MS1 (1)
- MS2 (2)
- MS3 (3)
- MS4 (4)
- Off-cycle or gap year (5)

Q12 Have you completed any clinical rotations at this point in your training? (Foundations of Doctoring preceptorship not included)

- Yes (1)
- No (2)
