# Supplementary material for: What Happened and Why: Responding to Racism, Discrimination, and Microaggressions in the Clinical Learning Environment
Source: MedEdPORTAL. 2022 Nov 1;18:11280. doi: 10.15766/mep_2374-8265.11280 (PMC9622434; doi:10.15766/mep_2374-8265.11280)
Supplement: Supplementary file 1 — Facilitator Guide.docxStudent Guide.docxRDM Faculty Development.pptxGuide for Implementation.docxPreworkshop Survey.docxPostworkshop Survey.docx [file mep_2374-8265.11280-s001.zip › F. Postworkshop Survey.docx]


What Happened and Why:

Responding to Racism, Discrimination, and Microaggressions in the Clinical Learning Environment

TRAINING CURRICULUM: **Workshop Post Survey**

Q1

Thank you for participating in the workshop!

In order to collect data anonymously in a way that we can combine data from the pre-and post-session survey as well as future data that will be collected at a later point in time, we ask that each participant create a unique identifying code.


Please list the two digits for your birth month followed by your last two social.

For example, if you were born in January and your social ends in 56 - enter 0156.


After all data is collected and linked your entered ID will be replaced with a unique random study-id so that no link to your unique identifiers will remain.

________________________________________________________________

________________________________________________________________

________________________________________________________________

________________________________________________________________

________________________________________________________________

Now that you have completed the workshop, take a moment to reflect on things that you learned and the scenarios presented. We will repeat a few questions that you answered at the start of the workshop and some that will be new to you. The working definition of micro-aggression that we will be using during this workshop is: A brief and commonplace daily verbal, behavioral, or environmental indignities, whether intentional or unintentional, that communicate hostile, derogatory, or negative slights and insults toward marginalized groups of people.

Q2 Have you yourself experienced or witnessed an instance of micro-aggression in a clinical setting during your time as a student?

- Yes (1)
- Maybe (2)
- No (3)

Free Response: If you answered yes to the above please add additional details about your experience.


Q3 How many times have you witnessed or experienced RDM instances directed toward yourself or others?

|  | Never (0) | Once (1) | More than once (2+) |
| --- | --- | --- | --- |
| Yourself (1) |  |  |  |
| Another student or member of the medical team (2) |  |  |  |
| The Patient (3) |  |  |  |

Q4 If you answered yes to any of the above, who was responsible for the micro-aggression in the scenario? Select all that apply.

- Myself (1)
- Attending (2)
- Fellow (3)
- Resident (4)
- Intern (5)
- Medical Student (6)
- Another health professions student (7)
- Another health professional (e.g., nurse, physical therapist, pharmacist) (8)
- Patient (9)
- Other (10) ________________________________________________

Q5 Please indicate your level of agreement with the following statements:

|  | Strongly Disagree (1) | Somewhat disagree (2) | Neither agree nor disagree (3) | Somewhat agree (4) | Strongly agree (5) |
| --- | --- | --- | --- | --- | --- |
| I am aware of different strategies to deal with RDMs aimed at other members of the medical team (1) |  |  |  |  |  |
| I am aware of different strategies to deal with RDMs aimed at myself (2) |  |  |  |  |  |
| I am aware of strategies to deal with RDMs aimed at patients, family-member, or other support person(s) (3) |  |  |  |  |  |
| I feel confident in applying communication strategies to deal with RDMs in the clinical learning environment (4) |  |  |  |  |  |

Q6 Please answer each of the following questions based on your current level of comfort for addressing instances of RDMs

|  | Strongly Disagree (1) | Somewhat disagree (2) | Neither agree nor disagree (3) | Somewhat agree (4) | Strongly agree (5) |
| --- | --- | --- | --- | --- | --- |
| I am comfortable addressing RDMs aimed at others (1) |  |  |  |  |  |
| I am comfortable addressing RDMs aimed at myself (2) |  |  |  |  |  |
| I am comfortable addressing RDMs aimed at patients (3) |  |  |  |  |  |
| I am comfortable addressing RDMs I have committed myself (4) |  |  |  |  |  |

Q7 Based on what you learned today, please rank the following tactics discussed based on which you think you will be most likely to use in the future

- Direct
- Distract
- Delegate
- Defer
- Display Discomfort
- Debrief

|  | Very Unlikely (1) | Somewhat Unlikely (2) | Neither Likely nor Unlikely (3) | Somewhat Likely (4) | Very Likely (5) |
| --- | --- | --- | --- | --- | --- |
| Direct (1) |  |  |  |  |  |
| Distract (2) |  |  |  |  |  |
| Delegate (3) |  |  |  |  |  |
| Defer (4) |  |  |  |  |  |
| Debrief (5) |  |  |  |  |  |

Q8 Do you have a scenario that you experienced that you think may make a good case for future sessions?

- Yes
- No

Q9 If you are comfortable sharing, please briefly describe the scenario.
