# Supplementary material for: What Happened and Why: Responding to Racism, Discrimination, and Microaggressions in the Clinical Learning Environment
Source: MedEdPORTAL. 2022 Nov 1;18:11280. doi: 10.15766/mep_2374-8265.11280 (PMC9622434; doi:10.15766/mep_2374-8265.11280)
Supplement: Supplementary file 1 — Facilitator Guide.docxStudent Guide.docxRDM Faculty Development.pptxGuide for Implementation.docxPreworkshop Survey.docxPostworkshop Survey.docx [file mep_2374-8265.11280-s001.zip › A. Facilitator Guide.docx]

What Happened and Why:

Responding to Racism, Discrimination, and Microaggressions in the Clinical Learning Environment

TRAINING CURRICULUM: **Facilitator Guide**

**Learning Objectives:**

1. Recognize racism, discrimination and microaggressions (RDM) that medical students may experience in the clinical setting.
2. Identify preferred strategies when faced with bias-related conflict.
3. Practice mitigation strategies when witnessing RDM.
4. Discuss the implications of discriminatory and/or biased acts on ourselves, our peers, and our patients.

**Session Preparation:**

For faculty:

1. Review this facilitator guide
2. Review the facilitator development PowerPoint or attend a facilitator faculty development session

**Agenda:**

| **Estimated duration** | **Session Component** | **Page #** |
| --- | --- | --- |
| 15 minutes | Introduction and Overview | 3 - 5 |
| 20 minutes | Assessing Current Response Toolbox Using Clinical Scenarios | 6 |
| 70 minutes | Skills Practice | 6 - 11 |
| 10 minutes | Debrief: Moving Forward After Encountering RDM in Clinical Settings | 12 |
|  | Case resolutions (only in facilitator guide) | 13 - 14 |

**Detailed Agenda:**

*Identify and Respond to Bias, Discrimination and Microaggressions in the Clinical Setting- Scenario-Based Strategies to Respond Safely*

**5 Minutes: Pre-session survey and Introduction to Group and Session**

Please provide students 2 minutes to complete the optional pre-session survey (accessible via QR code in their student guide).

To introduce the group, you may begin by saying something like: “let’s go around and introduce ourselves as a group. Tell us your name, pronouns, your hometown, and what you are most excited about for your clinical year.”

**Ground Rules for Discussion in Small Groups**

It is important to set ground rules for the conversation to create a brave space, with full and equitable participation of people from all social identity groups in a society that is mutually shaped to meet their needs. We are hoping to create a space where everyone actively participates. We encourage you to review the recommended community norms with the group and adapt any to meet the groups’ needs.

- Confidentiality: Respect individual’s privacy by not sharing their names or identifying details
- Be open
- Diversity of opinions and ideas are valued
- Honor and respect your peers
  - One person speaks at a time
  - Respect other’s views even if you disagree, question others’ views if you feel offended
  - There are no right or wrong answers
  - Use “I” statements
  - No personal attacks
- Argue about the issues/ideas, not about identities and experiences.

If one of the ground rules is broken and/or another challenging moment were to arise, as a facilitator, pausing and checking-in with the team is recommended. An example phrase you may consider using is: “hey everyone, you are all doing such a great job [being open]. These are challenging conversations and I do want to pause and remind us of one of our ground rules, which is that [there are no right or wrong answers.]

"**10 min: Session Overview: Defining Terms and Goals**

**Review Definitions and Goals of the Session:**

Microaggressions are subtle statements and behaviors that communicate denigrating messages to people who identify with minoritized or marginalized social groups*.* These actions perpetuate stereotypes and devalue groups. In the learning environment, the cognitive burden and increased workload for the recipient impact academic and work performance and negatively impact the entire team. As a learner, your work environment should be safe and respectful. By the end of this session, you will have a formal framework of response mechanisms to help you navigate acts of RDM. We will develop this through skills practice.

**Review mitigation strategies to address RDM (6 D’s):**

Please ask the students to read the following descriptions and examples to become familiar with the meaning of each of these response strategies and roles as we will be referencing them throughout the session. The first 4 D’s describe options for how to respond to racism, microaggressions, and acts of bias. The final 2 D’s are responses we encourage you to use with every incident.

**Response Strategies**

1. **Direct:** Directly intervene and address the situation in the moment. Defy and challenge the person to clarify their statement or their action. Exploratory Questions can be a powerful tool. Stating “How do you mean that?” or “I’m curious what you mean by that” gives people a chance to check themselves and it gives you the opportunity to better gauge that individual’s intent.
   1. As a recipient:
      1. “What did you just say?”
      2. “I actually am not “X.” I am the medical student caring for you.”
   2. As a bystander:
      1. “What you just said is not ok.”
      2. “What did you mean when you said…”
2. **Distract:** For those who are not in supervisory roles, or when interacting with someone with more “power” in traditional hierarchies, a tactic can be to redirect the focus or change the subject to a relevant topic.
   1. As a recipient:
      1. “Oh, my pager is going off.”
      2. “Did you hear that alarm?”
   2. As a bystander:
      1. “Can I ask a question about?”
      2. Perform a distracting action (drop something, spill a drink) to shift focus
3. **Delegate:** Speak to someone else to discuss appropriate next steps. You can leave the environment, and subsequently, ask a supervisor to address the situation.
   1. As a recipient
      1. “I am not comfortable with your comments. I am going to consult with my supervising doctor to ensure that you get the appropriate care.”
   2. As a bystander
      1. “What you just said is not an appropriate way to interact with our team members. The attending physician will be in to speak to you.”
4. **Defer:** delay your response. Consider waiting for a few hours or a day to better reflect on the situation or talking to someone before reporting the event. You can disregard the action entirely.
   1. As a recipient:
      1. When you chose to delay, identify when you will make time to reflect and who you will talk to.
      2. If you disregard it, how would you feel if you decide never to talk about it?
   2. As a bystander:
      1. “Are you ok, I heard (or saw) that, and I’m sorry I didn’t say anything in the moment, but I wanted to check in with you.”
      2. If you disregard it, how would you feel as a bystander who never said anything?
5. **Display discomfort:** Use body language to show you noticed something wrong or inappropriate. Furrow your brow. Frown. Shake your head. Do anything EXCEPT pretend like nothing happened.
   1. Another way to show discomfort is using an Awkwar”D” silence. If you experience or witness bias in a conversation, stop talking, create an awkward pause and remain quiet until the other person acknowledges what they said.
6. **Debrief:** Arguably the most important step and should be done every time. Rarely, the actual upstanding part goes smoothly. The debrief is everyone’s chance to reflect on the moment and support one another. Let everyone know how you felt and ask how others are feeling.

Before we begin applying these response strategies, here are a few things that you should keep in mind.

1. As a medical student or physician, you can appropriately remove yourself from the presence of a patient that disrespects you. If able, immediately consult with a supervising attending/resident who has the responsibility to address the patient directly.
2. The AMA Code of Medical Ethics states: “Trust can be established and maintained only when there is mutual respect. Therefore, in their interactions with patients, physicians should:
   1. Recognize that derogatory or disrespectful language or conduct can cause psychological harm to those they target.
   2. Always treat their patients with compassion and respect.
   3. Terminate the patient-physician relationship with a patient who uses derogatory language or acts in a prejudicial manner only if the patient will not modify the conduct. In such cases, the physician should arrange to transfer the patient’s care”.
3. We also understand that STOPPING a situation immediately is more difficult when there is a power differential. In those cases, even if you do not address the situation at the moment, the act of reporting can be a powerful tool for positive change.
4. We STRONGLY ENCOURAGE you to talk to anyone you feel comfortable with any time you experience something that makes you feel distressed. Even if you do not feel comfortable reporting. This training was designed to empower you to better manage these difficult experiences.
5. The incidents discussed today reflect real case scenarios experienced by students in the classes of 2021 and 2022 in the CUSOM. {NOTE: we recommend institutions use examples from their own learners for these scenarios}. Please maintain the confidentiality of this session and respect individuals’ privacy by not sharing names and identifying details from these cases and your small group’s discussions.

**20 min: Assessing Current Response Toolbox Using Clinical Scenarios**

Now that these response strategies and roles have been introduced, we would like you to reflect on how you would currently react to situations of RDM in the clinical setting. You do not need to use the response strategies discussed above. Students should divide into 2 groups and each group should discuss how they would respond in the following roles given the following short scenarios:

1. Recipient: Respond as you might if you were the student who is on the receiving end of the instance of racism, discrimination, or microaggression described in the case.
2. Bystander: Describe what you would do as a bystander witnessing the instance of racism, discrimination, or microaggression described in the case.

Case 1: group 1 discusses how they would respond as a recipient; group 2 discusses how they would respond as a bystander

Case 2: group 2 discusses how they would respond as the recipient; group 1 discusses how they would respond as a bystander.

Students should explore why they think they would respond in that way and how that reflects their past experiences. They should also identify how their reaction matches the response strategies introduced. *Discussion questions are listed below to facilitate this conversation.*

**Case 1:**

- **During my shift at my surgery preceptorship, I had a henna tattoo on my hand. When we were in the surgery lounge, the PA who was assisting in the upcoming surgery told me that I had to “wash that stuff off or people are gonna think you’ve got poop on your hands.”**

**Case 2:**

- **As I move toward the patient to shake hands before I leave the room, the patient grabs my hand and says in a friendly manner “I want a handshake like you do in the ghetto.” I am a Black medical student.**

Discussion questions:

- *How would you respond?*
- *Why would you respond that way?*
- *What past experiences influenced the way you chose that response?*
- *Did your response align with any of the response strategies above?*
- *How would your response have changed if you were in a different role?*

**70 min: Skills Practice**

Students will practice mitigation strategies and responses. Assign each student a letter. Based on their letter, the students will take the following roles for each case (according to the matrix chart below). Using their assigned responses and roles, students will rehearse what they might say in the situation described by the case. For the purpose of this exercise, the response of deferring will not be practiced. Remind the students that this is skills practice, meaning that they should practice saying the actual words they would use and not talk about what they might say. This matrix allows up to 6 students to practice responses for each case however, if there are only 5 students in your group, you can just assign letters A-E.

|  | A | B | C | D | E | F |
| --- | --- | --- | --- | --- | --- | --- |
| Case 1 | Direct-recipient | Direct-bystander | Delegate- recipient | Delegate-bystander | Distract-bystander | Debrief-bystander |
| Case 2 | Distract-bystander | Debrief- bystander | Direct-recipient | Direct-bystander | Delegate- recipient | Delegate-bystander |
| Case 3 | Delegate- recipient | Delegate-bystander | Distract-bystander | Debrief- bystander | Direct-recipient | Direct-bystander |
| Case 4 | Direct- bystander | Direct- recipient | Debrief-bystander | Direct- recipient | Direct-bystander | Delegate- recipient |

**Case 1:**

- Your clinic preceptor during a clinical rotation introduces you to patients as “one of the office girls.” On a different day, clinic staff tells you that you need to shave your legs a little better because the current state of your legs is unprofessional.

**Case 2**

- On my surgery rotation, a nurse asked me where my last name was from and I told them I’m Nigerian. The nurses and attendings during the surgery proceeded to laugh at me and say that they’re surprised my English is so good. Asked me if I was a citizen or just visiting. And they asked if I eat raw fish heads and rodents.

**Case 3**

- During my psychiatry rotation, I had an attending that was outwardly transphobic. Two of the patients I was following happened to be trans. In the first instance, my attending said regarding a trans woman was that “it was hard to tell whether he or was a girl or a boy” and then laughed. In the second instance, it was this young, suicidal trans man who was being admitted. My attending referred to our patient as “he/she” throughout our discussion of care and then made jokes about intentionally using “he/she” to refer to him. This hurt deeply because I was saddened by how she was discriminating against our patients when they were in serious need of psychiatric care. It also was incredibly hurtful to me as a trans man.

**Case 4**

- On my first day of clinical rotations as a third-year medical student, I was called an “n-word” followed by the “c-word"(please do not verbalize the following full words out loud: “ni**er c*nt”) by a white male patient; additionally, when my entire team was introduced as doctors, the patient said that “she is probably not a doctor” and he looked directly at me.

*Please share details of the resolutions of the cases with the students (listed on following pages)*

**10 min: Moving Forward After Encountering RDM in Clinical Settings**

Debrief: Talking about the event with someone you trust is as important, if not more important than, responding to the incident in the moment. For those who have experienced acts of racism, discrimination, and bias, debriefing can serve several roles—validation that what they experienced was real, validation that their emotional response (whatever it may be) is normal, recognition that what just happened to them was not acceptable and not their fault and allowing them to process the situation.

**Steps to take:**

1. TALK: Reach out to peers, advisors, mentors, or to the {insert relevant campus office/campus level resource for specific available options in the setting of crises/mental health challenges} for immediate support.
2. How to TALK about the event:
   1. “I am not sure what happened, but I need to talk through this scenario with you.”
   2. “I feel unsure about how to address/handle this situation. Could we talk through what you would do in this case?”
   3. “Could we possibly debrief a/the situation that just happened?”

**Additional resources you may access and/or share with another:**

1. In the case of a crisis, please consider texting the National Crisis Text Line: Text HOME to 741741 to reach a crisis counselor for further support/guidance
2. If you are struggling with mental health concerns, please consider reaching out to the Mental Health Services National Helpline at (800) 662-4357
3. For graduate students struggling with mental health concerns, please consider reaching out to the National Grad Crisis Line at 1-877-472-3257

**Conclusion:**

You control what each incident will mean for you. People from marginalized and intersectional identities are subject to biased expectations and evaluations daily. We hope all of you will now feel better equipped to respond to acts of racism, discrimination, and microaggressions for yourselves and others.

“Let protecting your joy be your greatest and most persistent act of resistance.”

*{Include campus resources for reporting and support here.}*

***At Conclusion of the Session, please encourage students to complete their post-session surveys***

**Thank you.**

**Authors:**

Helio V. Neves da Silva; Lauren M. Heery; William R. Cohen; Vikasini S. Mahalingam; Oluwatosin A. Adebiyi; Rita S. Lee, MD; Adom N Netsanet; Eniola A. Ogundipe; Yasmine Dakhama; Mary L. Wang; Michael Aaron Vrolijk, MD, MS, MA; Mackenzie W. Garcia; Jacqueline Ward-Gaines; Anna T. Neumeier

**Resolution of cases:**

**Case 1:**

Did you or anyone else say anything in response to these comments?

I reminded the patient that I was a third-year medical student at some appropriate time in the conversation. The patient responded favorably and appeared impressed. In response to being asked to shave my legs- I did not. I had hairy legs for the remainder of the term out of sheer defiance.

Did anyone else react? Nope

Did you report these comments to anyone or debrief with peers/friends/mentors? Not really. Only with one friend.

**Case 2:**

In response to the comments, I politely answered their questions. "I was born in the United States and have schooled here my entire life." "Where my family is from, we do not eat raw fish heads, nor do we eat rodents."

While the anesthesiologist was watching the whole thing, they did not speak up. No one else reacted to these comments. That particular strain of microaggressions did not occur again, but when the surgeon was rude to me on a different day, the anesthesiologist caught me in the scrub room and made a slight remark about how mean the surgeon is and not to take his behaviors personally.

I reported my experiences on that rotation to the Office of Diversity and Inclusion as well as the Surgery Clerkship Director. I am not sure what has come from my report.

**Case 3:**

No one said anything, but I could tell that a couple of us were uncomfortable in the room and no one was laughing along with her. It took me a while to debrief with anyone, but after some time I debriefed with friends. They encouraged me to report it – so I had a really good conversation with Dr. L about it, who has been incredibly supportive over a couple of times I experienced racist and transphobic treatment.

**Case 4:**

1. Did you or anyone else say anything in response to these comments?

In the room, no one (my attending or residents) directly told the patient anything in response to his inappropriate comments. I think the team was mostly scared about this patient’s behavior and thus did not tell the patient that he was being disrespectful. The patient had altered mental status, but that is not an excuse for racist comments. As an all-female team, I could possibly see the argument of not engaging in conversation with the altered patient for safety. However, my attending was the least helpful out of the entire room. After we left the patient’s room, the team looked confused but kept going along with the tasks of the day without even acknowledging what happened.

2. Did anyone else react?

My attending lacked the courage to take lead and control of the situation in the room despite being a senior physician at the hospital for many years. The residents did ask at the end of the day prior to dismissing me if I was okay (several hours later) and at that point, the damage was already done. The lack of debriefing directly after the incident exacerbated the harm because I felt my pain was unrecognized although I was visibly upset for the rest of the day.

3. Were you required to work with this patient again?

No, but I had the option to. I told my team that I did not want to see the patient again and my new attending said they were disgusted by the situation and offered to see the patient alone as to protect the team from additional trauma.

4. Did you report these comments to anyone or debrief with peers/friends/mentors?

I did not cry until I reached my car and I immediately called Dean Z and Dean J. I basically told everyone (friends, mentors, faculty members); this incident occurred in the same season as the BLM protests and advocacy. The hospital clerkship director reached out to me a day later and we met to discuss my experience. I think this was a shock to a lot of people because people forget that society’s injustices exist in the hospital setting too and I am not immune to racism just because I have a white coat.
