# Supplementary material for: What Happened and Why: Responding to Racism, Discrimination, and Microaggressions in the Clinical Learning Environment
Source: MedEdPORTAL. 2022 Nov 1;18:11280. doi: 10.15766/mep_2374-8265.11280 (PMC9622434; doi:10.15766/mep_2374-8265.11280)
Supplement: Supplementary file 1 — Facilitator Guide.docxStudent Guide.docxRDM Faculty Development.pptxGuide for Implementation.docxPreworkshop Survey.docxPostworkshop Survey.docx [file mep_2374-8265.11280-s001.zip › C. RDM Faculty Development.pptx]

## Slide 1
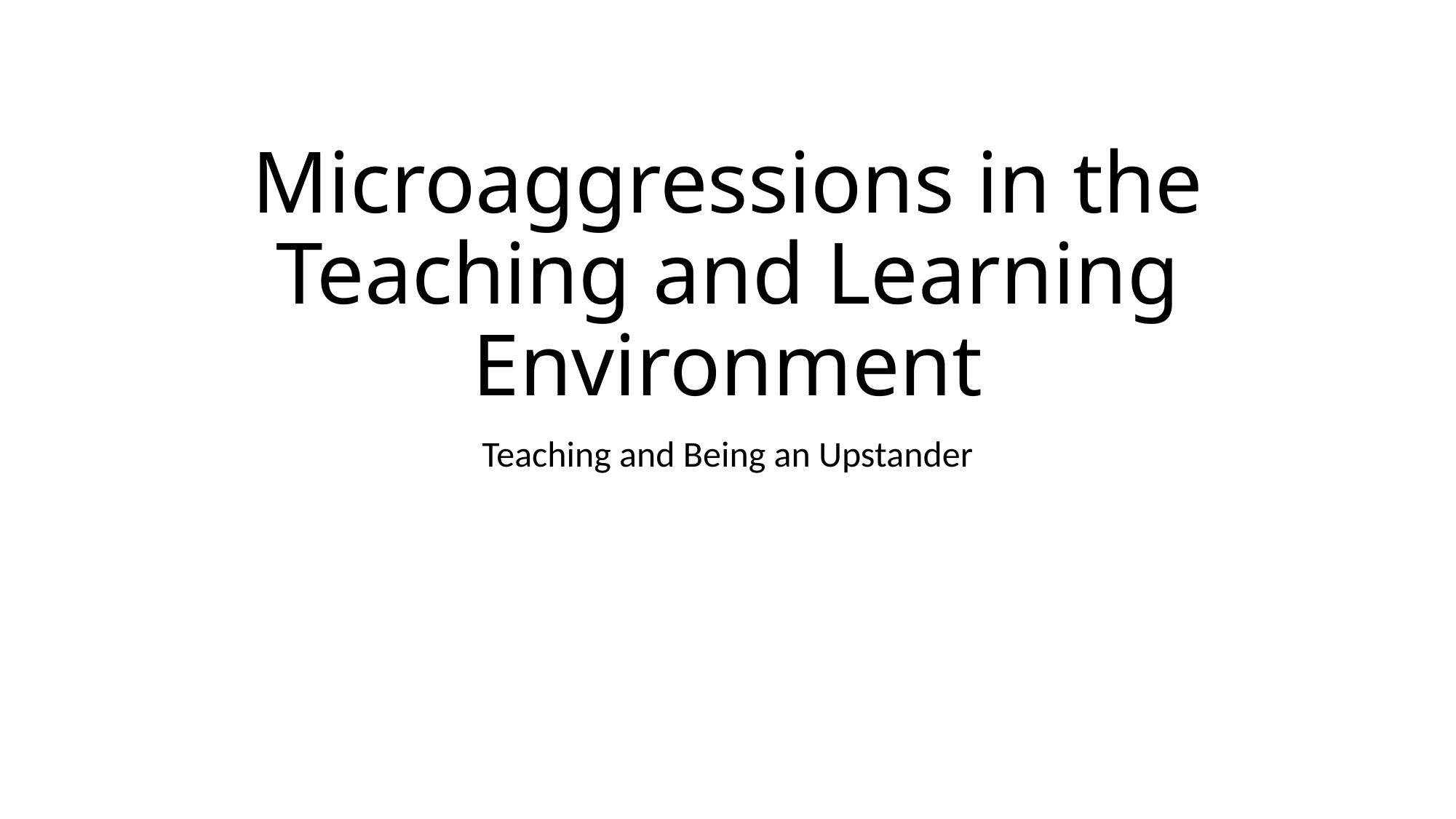

# Microaggressions in the Teaching and Learning Environment
Teaching and Being an Upstander

## Slide 2
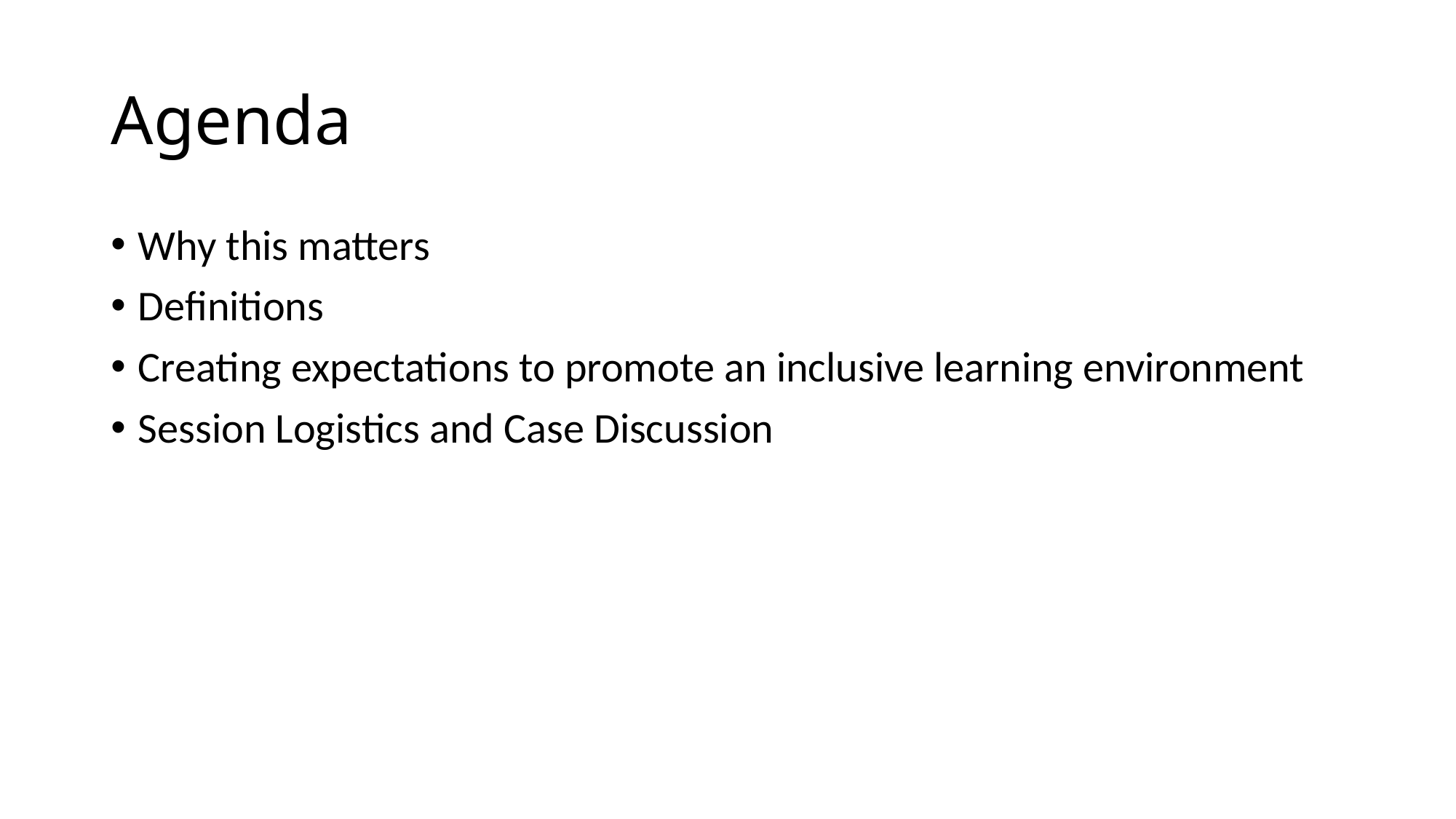

# Agenda
Why this matters
Definitions
Creating expectations to promote an inclusive learning environment
Session Logistics and Case Discussion

## Slide 3
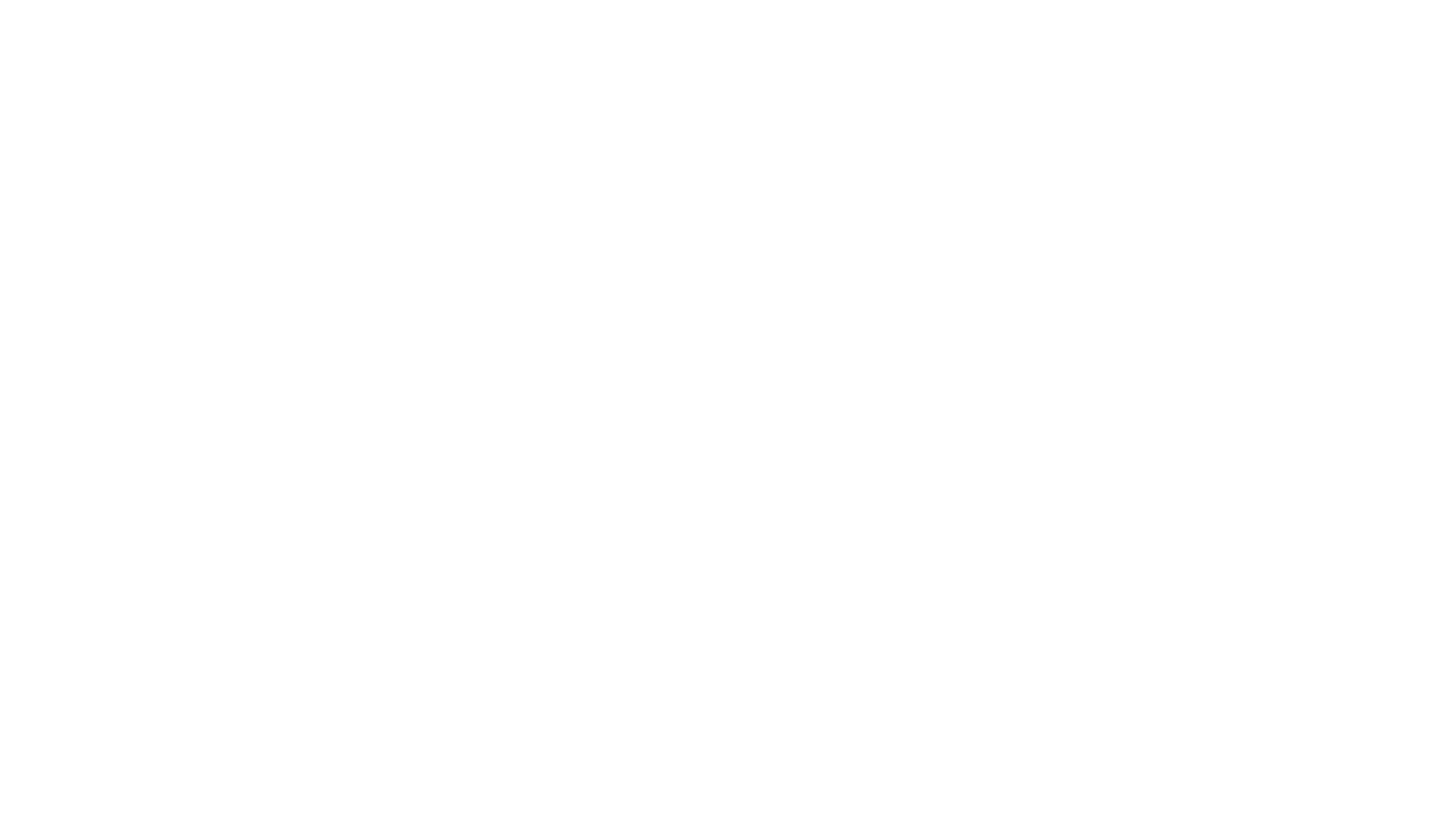

## Slide 4
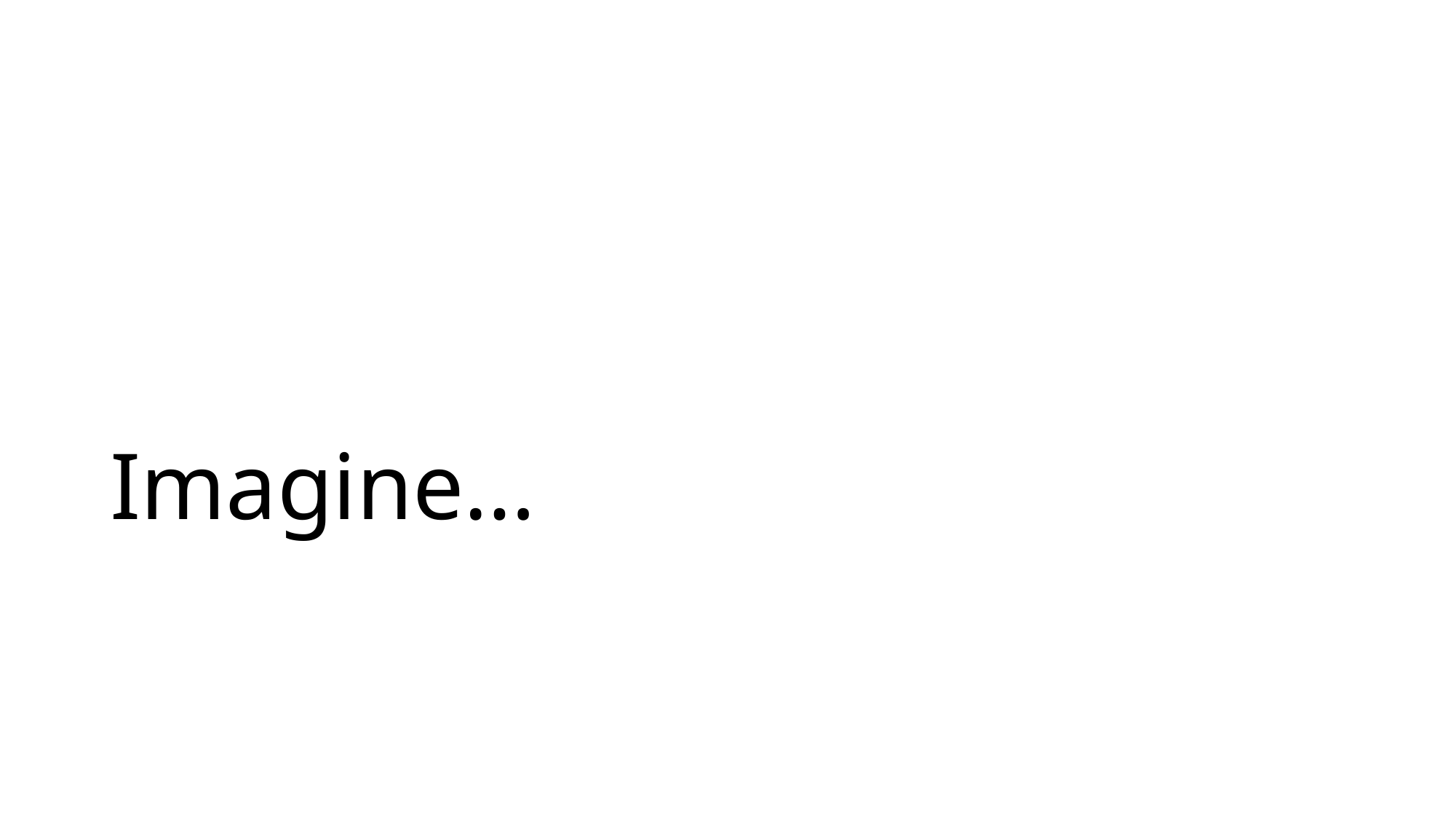

# Imagine…

## Slide 5
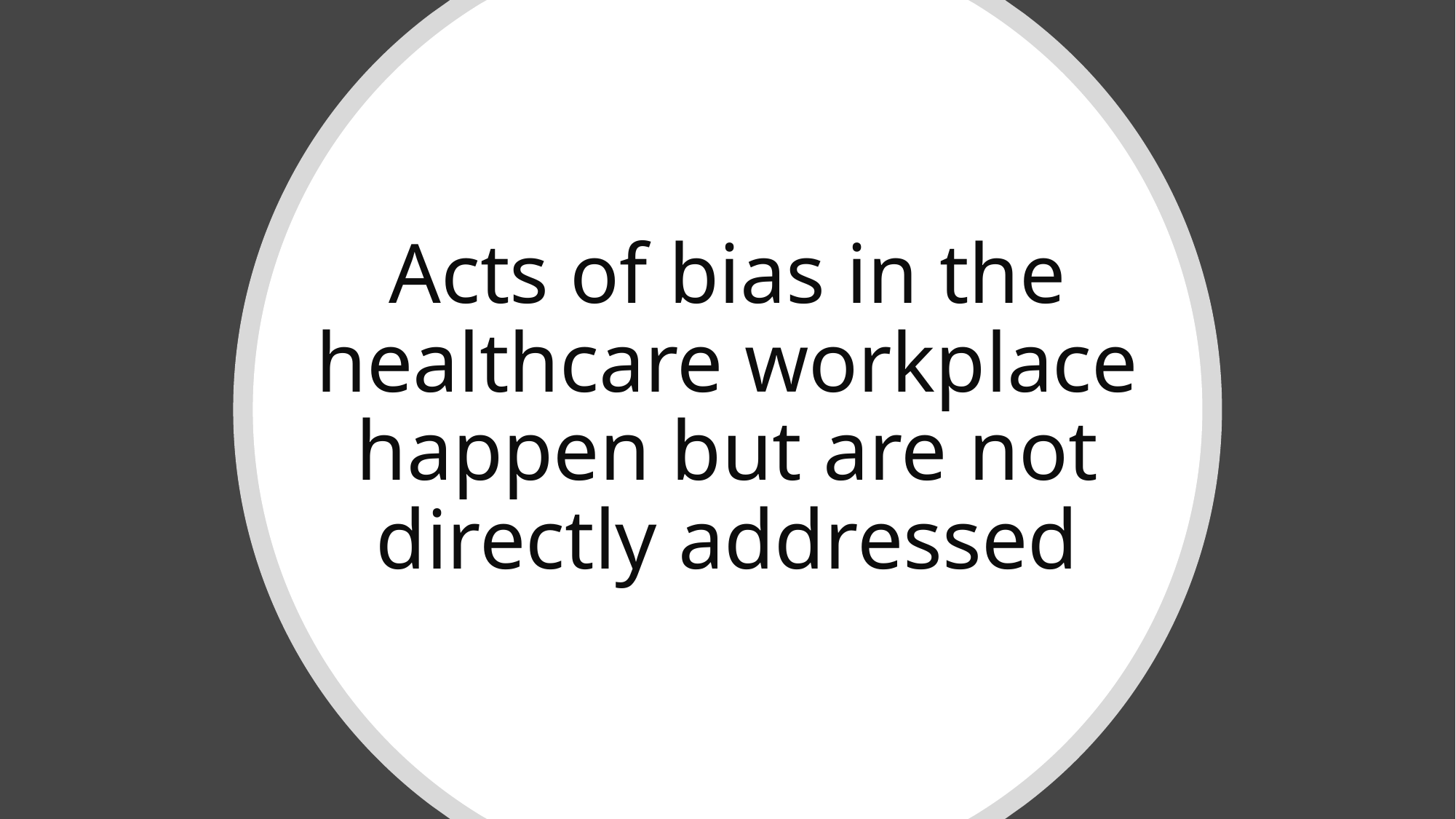

# Acts of bias in the healthcare workplace happen but are not directly addressed

## Slide 6
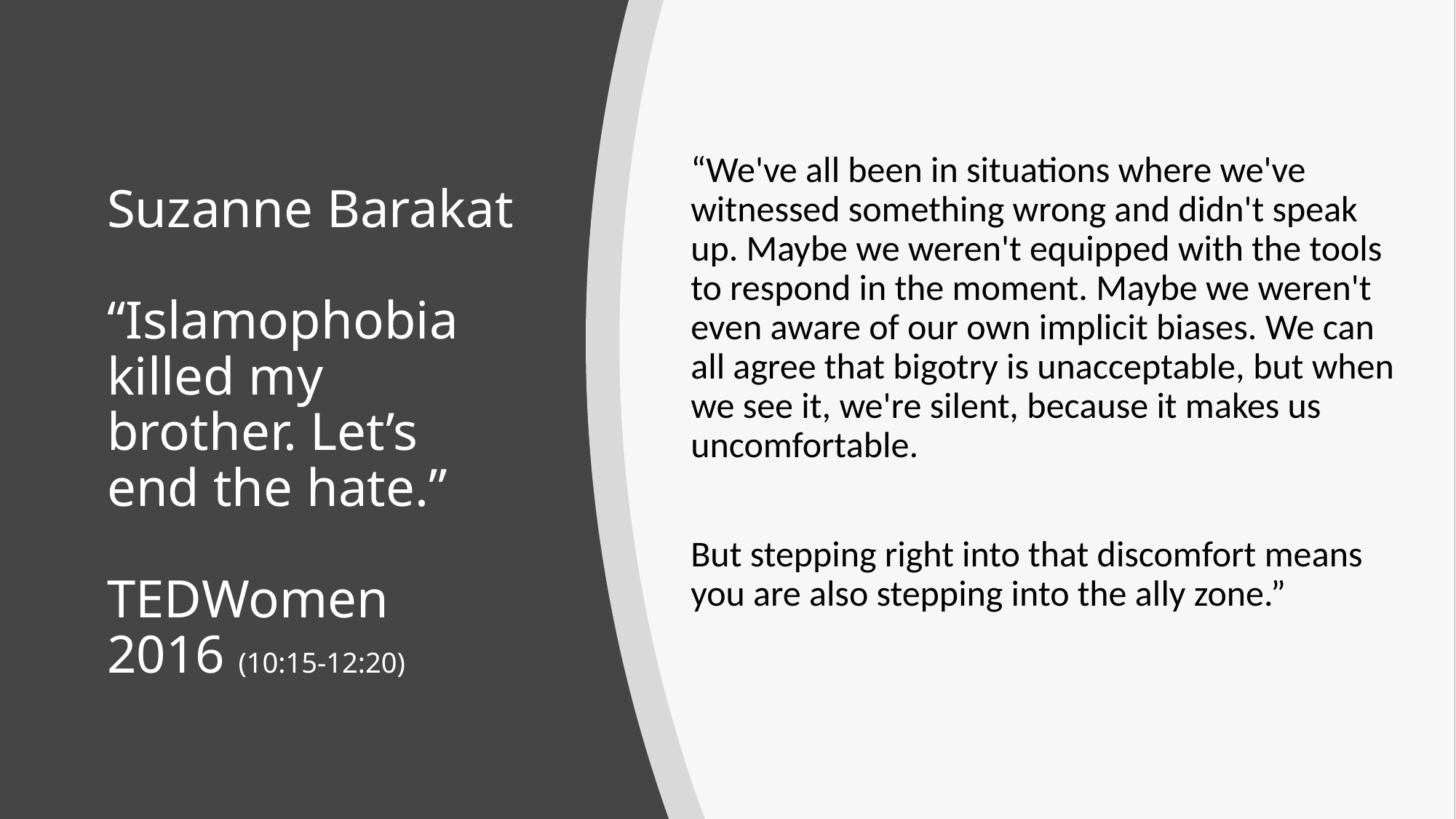

“We've all been in situations where we've witnessed something wrong and didn't speak up. Maybe we weren't equipped with the tools to respond in the moment. Maybe we weren't even aware of our own implicit biases. We can all agree that bigotry is unacceptable, but when we see it, we're silent, because it makes us uncomfortable.
But stepping right into that discomfort means you are also stepping into the ally zone.”
# Suzanne Barakat “Islamophobia killed my brother. Let’s end the hate.” TEDWomen 2016 (10:15-12:20)

## Slide 7
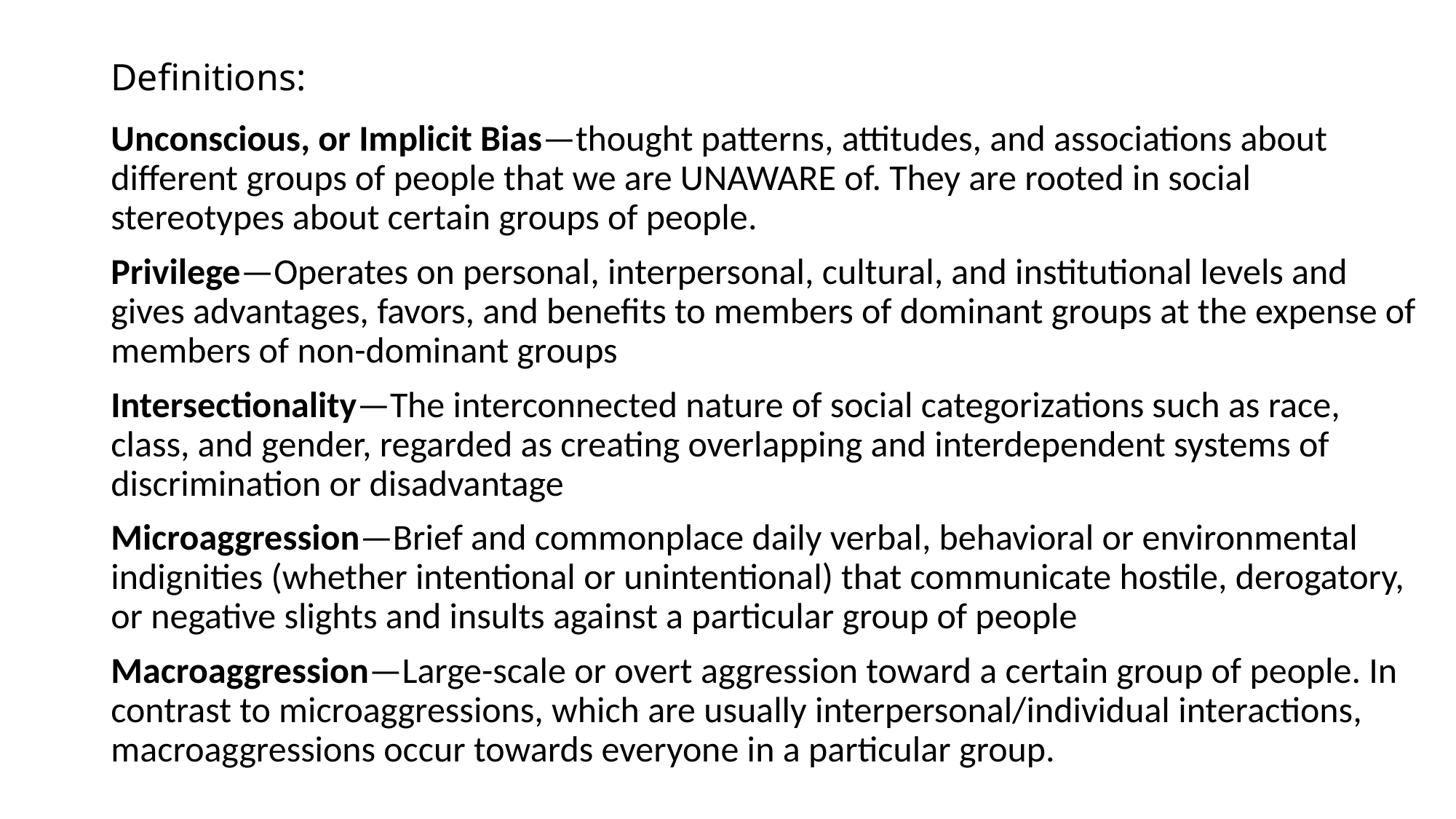

# Definitions:
Unconscious, or Implicit Bias—thought patterns, attitudes, and associations about different groups of people that we are UNAWARE of. They are rooted in social stereotypes about certain groups of people.
Privilege—Operates on personal, interpersonal, cultural, and institutional levels and gives advantages, favors, and benefits to members of dominant groups at the expense of members of non-dominant groups
Intersectionality—The interconnected nature of social categorizations such as race, class, and gender, regarded as creating overlapping and interdependent systems of discrimination or disadvantage
Microaggression—Brief and commonplace daily verbal, behavioral or environmental indignities (whether intentional or unintentional) that communicate hostile, derogatory, or negative slights and insults against a particular group of people
Macroaggression—Large-scale or overt aggression toward a certain group of people. In contrast to microaggressions, which are usually interpersonal/individual interactions, macroaggressions occur towards everyone in a particular group.

## Slide 8
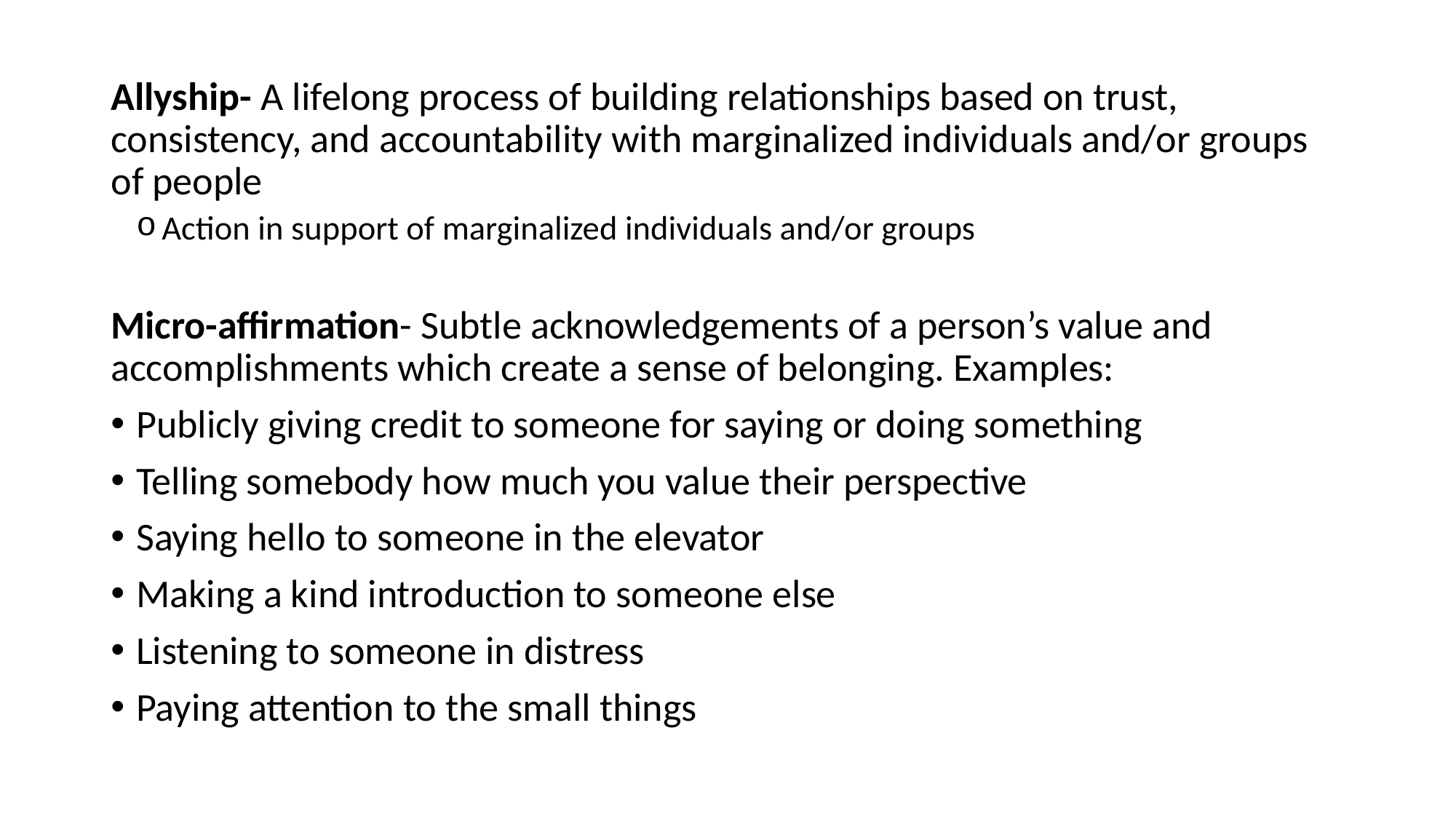

Allyship- A lifelong process of building relationships based on trust, consistency, and accountability with marginalized individuals and/or groups of people
Action in support of marginalized individuals and/or groups
Micro-affirmation- Subtle acknowledgements of a person’s value and accomplishments which create a sense of belonging. Examples:
Publicly giving credit to someone for saying or doing something
Telling somebody how much you value their perspective
Saying hello to someone in the elevator
Making a kind introduction to someone else
Listening to someone in distress
Paying attention to the small things

## Slide 9
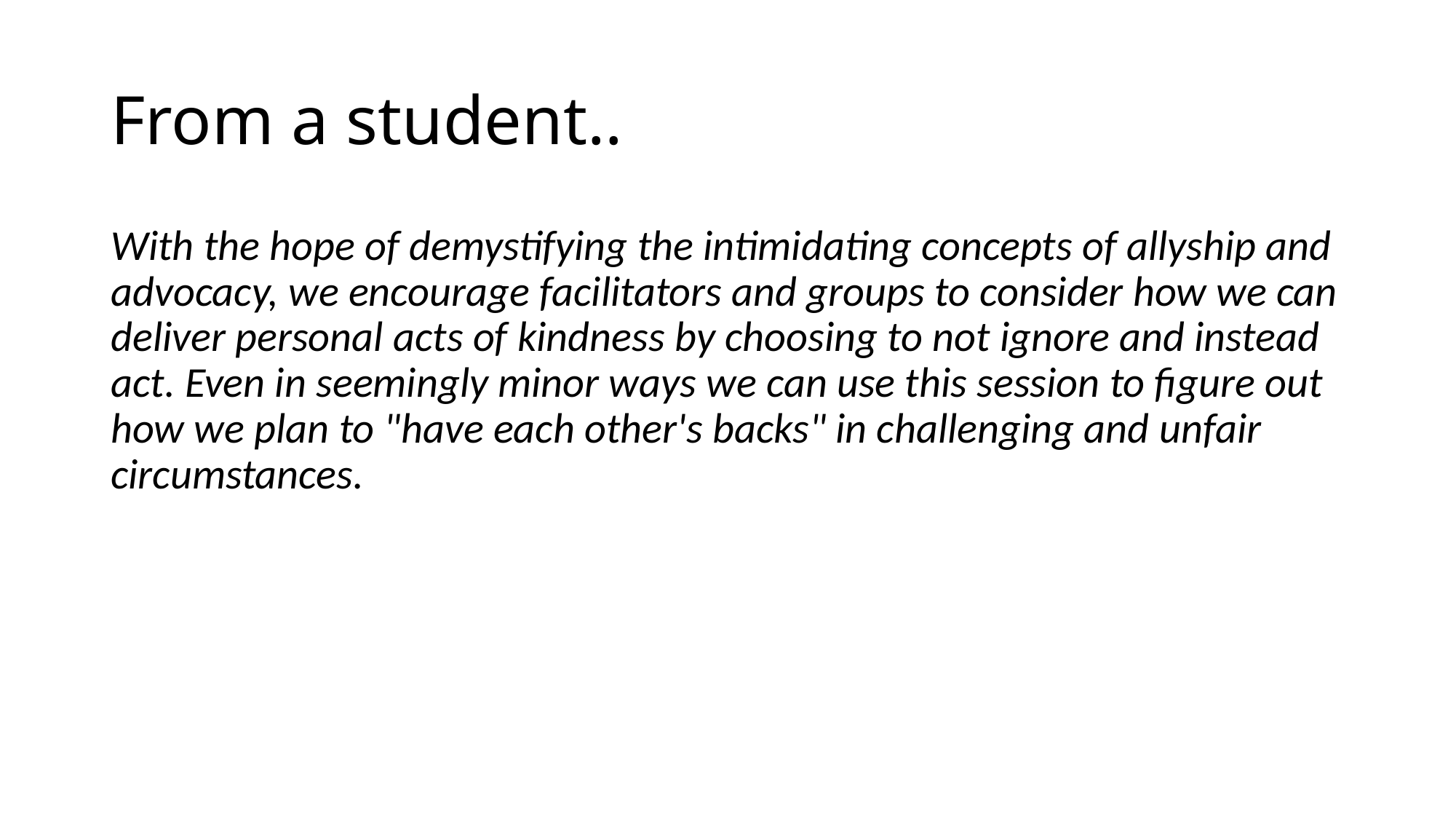

# From a student..
With the hope of demystifying the intimidating concepts of allyship and advocacy, we encourage facilitators and groups to consider how we can deliver personal acts of kindness by choosing to not ignore and instead act. Even in seemingly minor ways we can use this session to figure out how we plan to "have each other's backs" in challenging and unfair circumstances.

## Slide 10
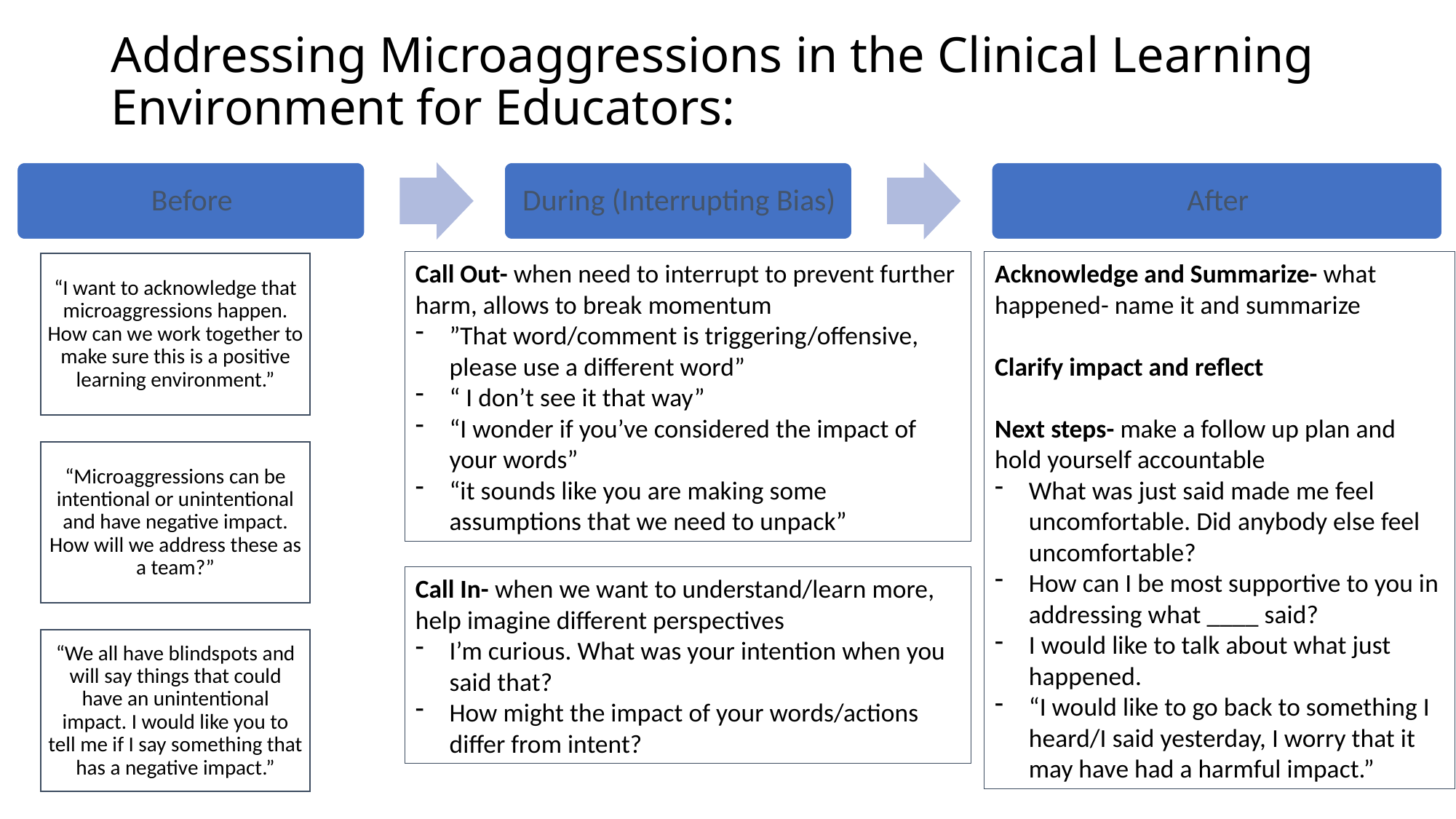

# Addressing Microaggressions in the Clinical Learning Environment for Educators:
Call Out- when need to interrupt to prevent further harm, allows to break momentum
”That word/comment is triggering/offensive, please use a different word”
“ I don’t see it that way”
“I wonder if you’ve considered the impact of your words”
“it sounds like you are making some assumptions that we need to unpack”
Acknowledge and Summarize- what happened- name it and summarize
Clarify impact and reflect
Next steps- make a follow up plan and hold yourself accountable
What was just said made me feel uncomfortable. Did anybody else feel uncomfortable?
How can I be most supportive to you in addressing what ____ said?
I would like to talk about what just happened.
“I would like to go back to something I heard/I said yesterday, I worry that it may have had a harmful impact.”
Call In- when we want to understand/learn more, help imagine different perspectives
I’m curious. What was your intention when you said that?
How might the impact of your words/actions differ from intent?

## Slide 11
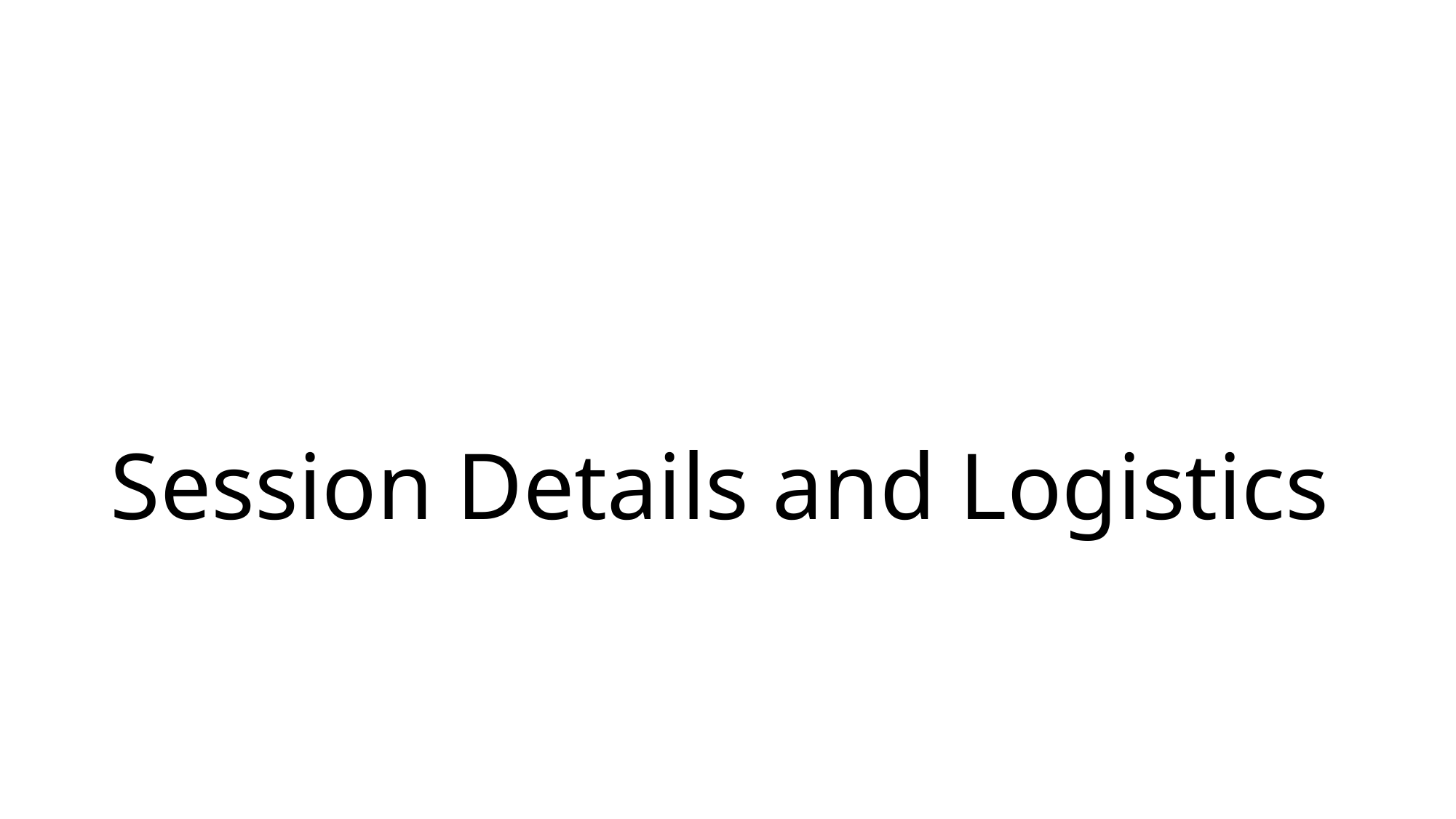

# Session Details and Logistics

## Slide 12
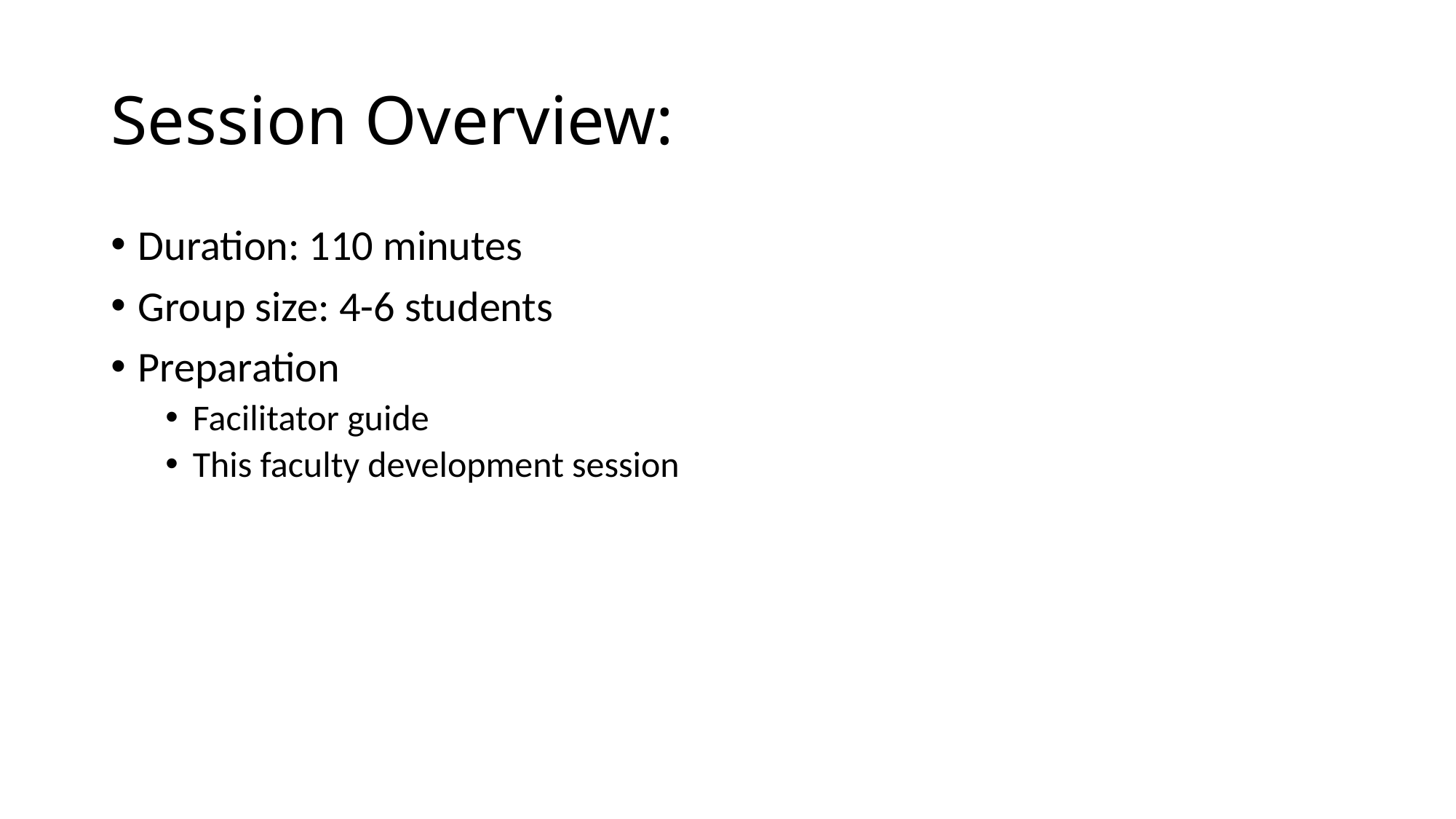

# Session Overview:
Duration: 110 minutes
Group size: 4-6 students
Preparation
Facilitator guide
This faculty development session

## Slide 13
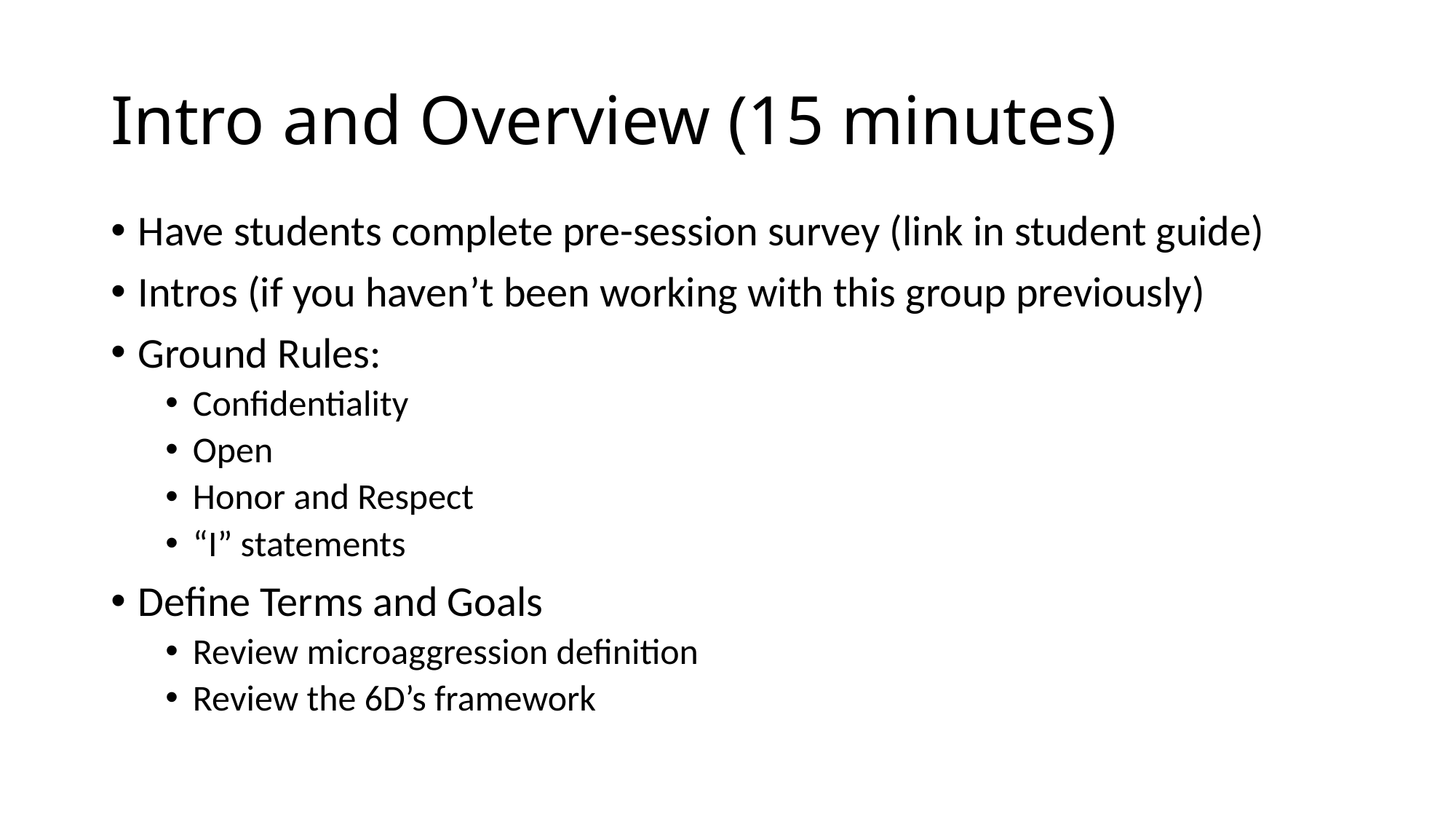

# Intro and Overview (15 minutes)
Have students complete pre-session survey (link in student guide)
Intros (if you haven’t been working with this group previously)
Ground Rules:
Confidentiality
Open
Honor and Respect
“I” statements
Define Terms and Goals
Review microaggression definition
Review the 6D’s framework

## Slide 14
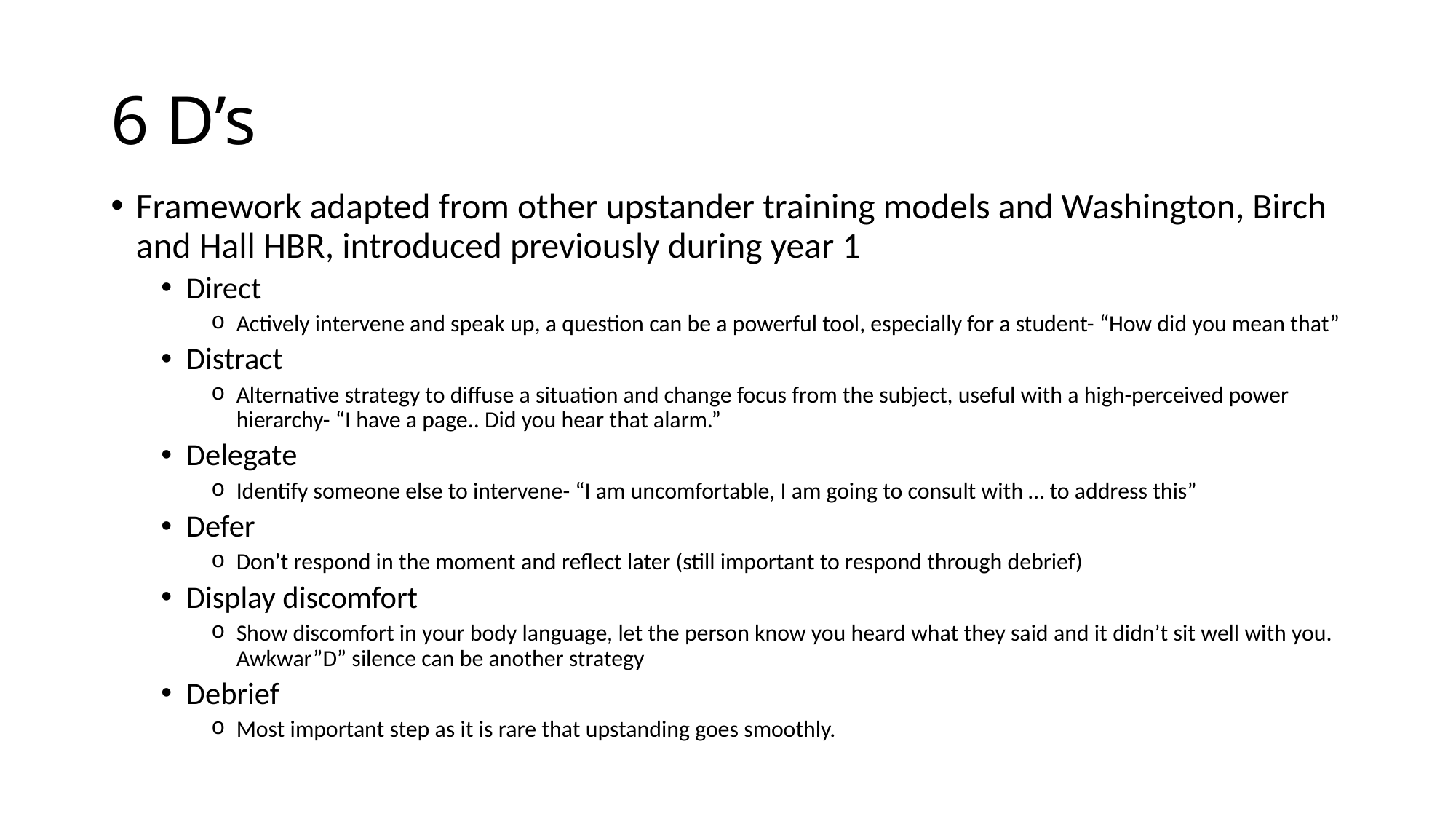

# 6 D’s
Framework adapted from other upstander training models and Washington, Birch and Hall HBR, introduced previously during year 1
Direct
Actively intervene and speak up, a question can be a powerful tool, especially for a student- “How did you mean that”
Distract
Alternative strategy to diffuse a situation and change focus from the subject, useful with a high-perceived power hierarchy- “I have a page.. Did you hear that alarm.”
Delegate
Identify someone else to intervene- “I am uncomfortable, I am going to consult with … to address this”
Defer
Don’t respond in the moment and reflect later (still important to respond through debrief)
Display discomfort
Show discomfort in your body language, let the person know you heard what they said and it didn’t sit well with you. Awkwar”D” silence can be another strategy
Debrief
Most important step as it is rare that upstanding goes smoothly.

## Slide 15
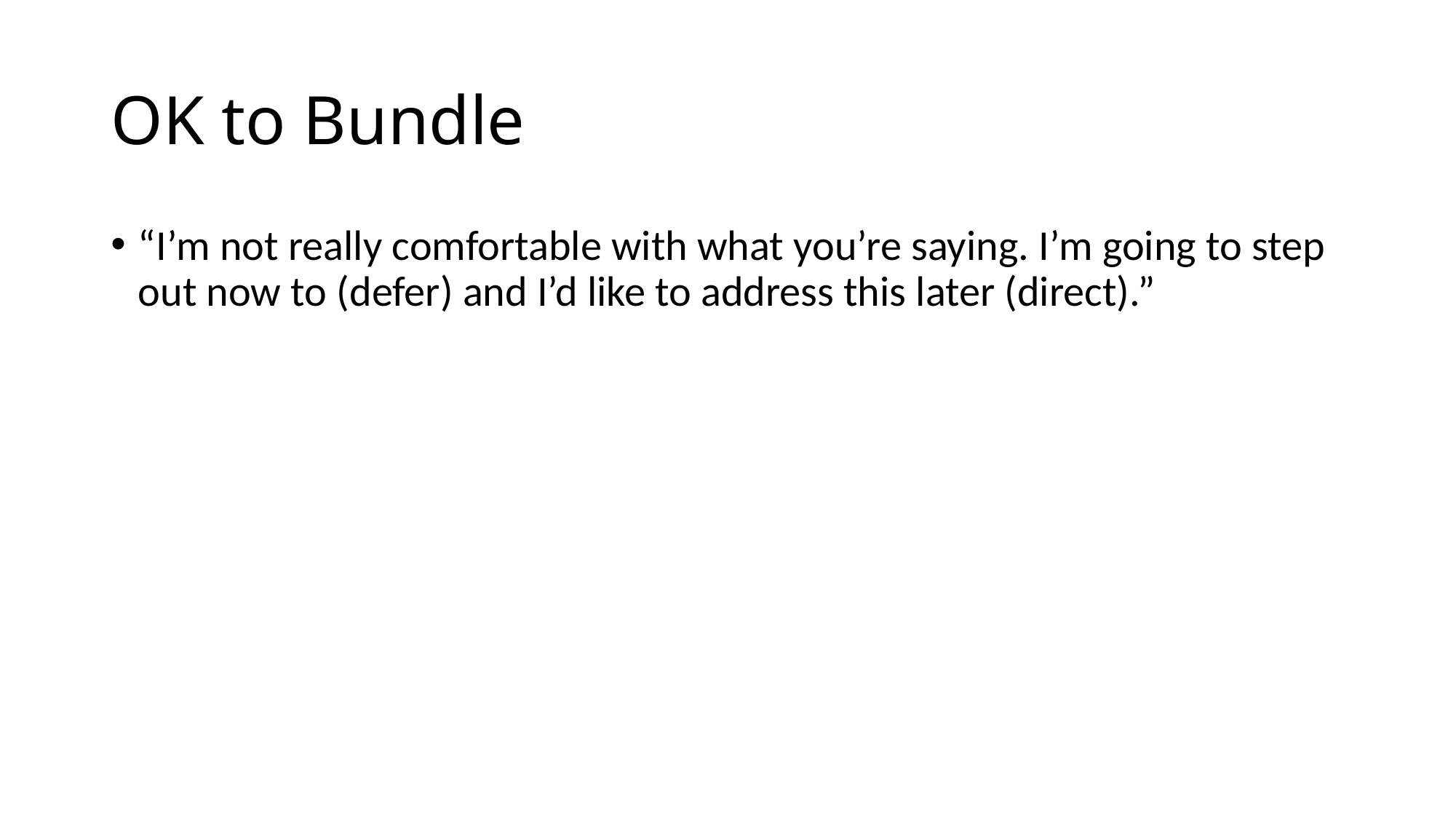

# OK to Bundle
“I’m not really comfortable with what you’re saying. I’m going to step out now to (defer) and I’d like to address this later (direct).”

## Slide 16
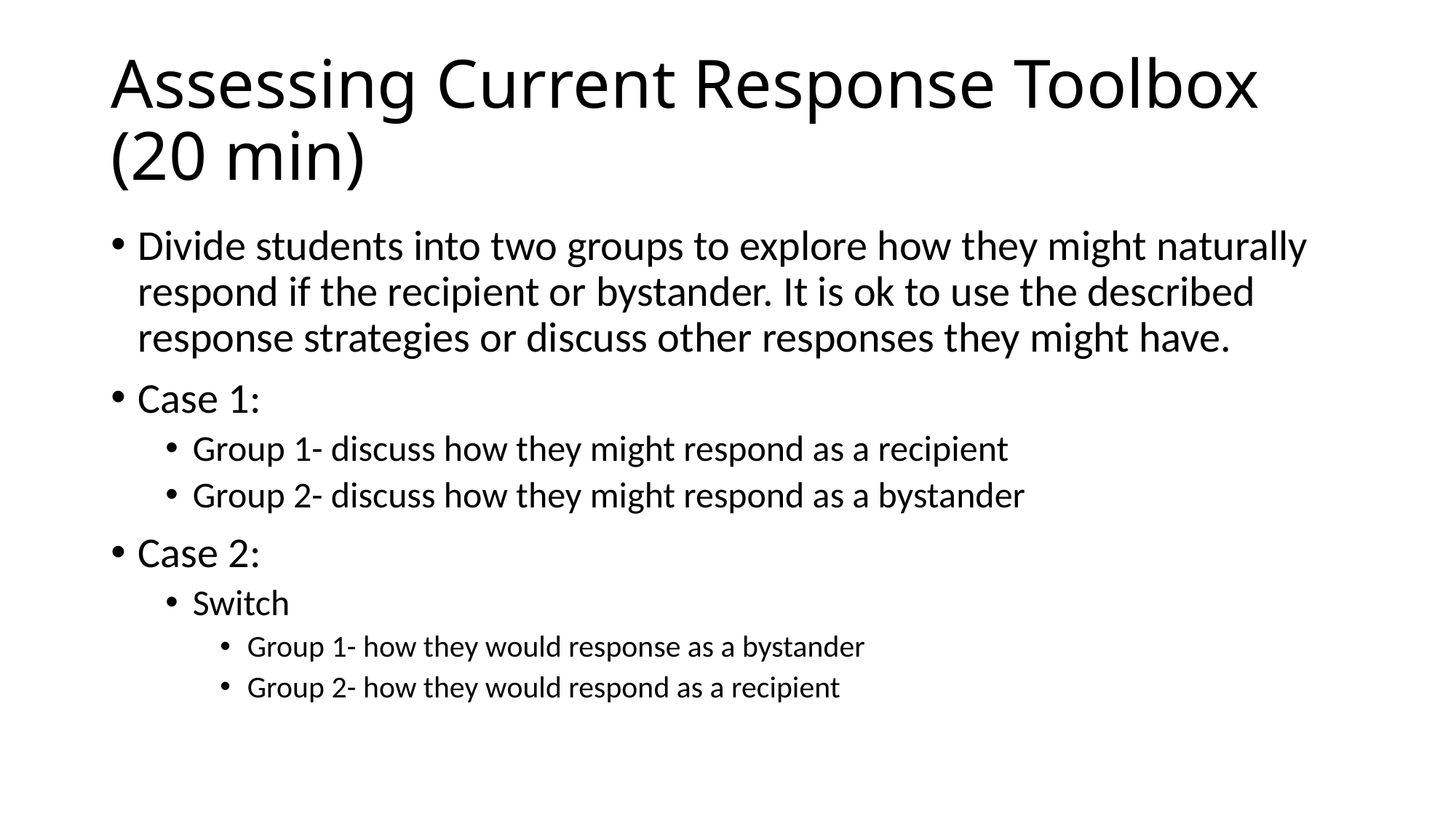

# Assessing Current Response Toolbox (20 min)
Divide students into two groups to explore how they might naturally respond if the recipient or bystander. It is ok to use the described response strategies or discuss other responses they might have.
Case 1:
Group 1- discuss how they might respond as a recipient
Group 2- discuss how they might respond as a bystander
Case 2:
Switch
Group 1- how they would response as a bystander
Group 2- how they would respond as a recipient

## Slide 17
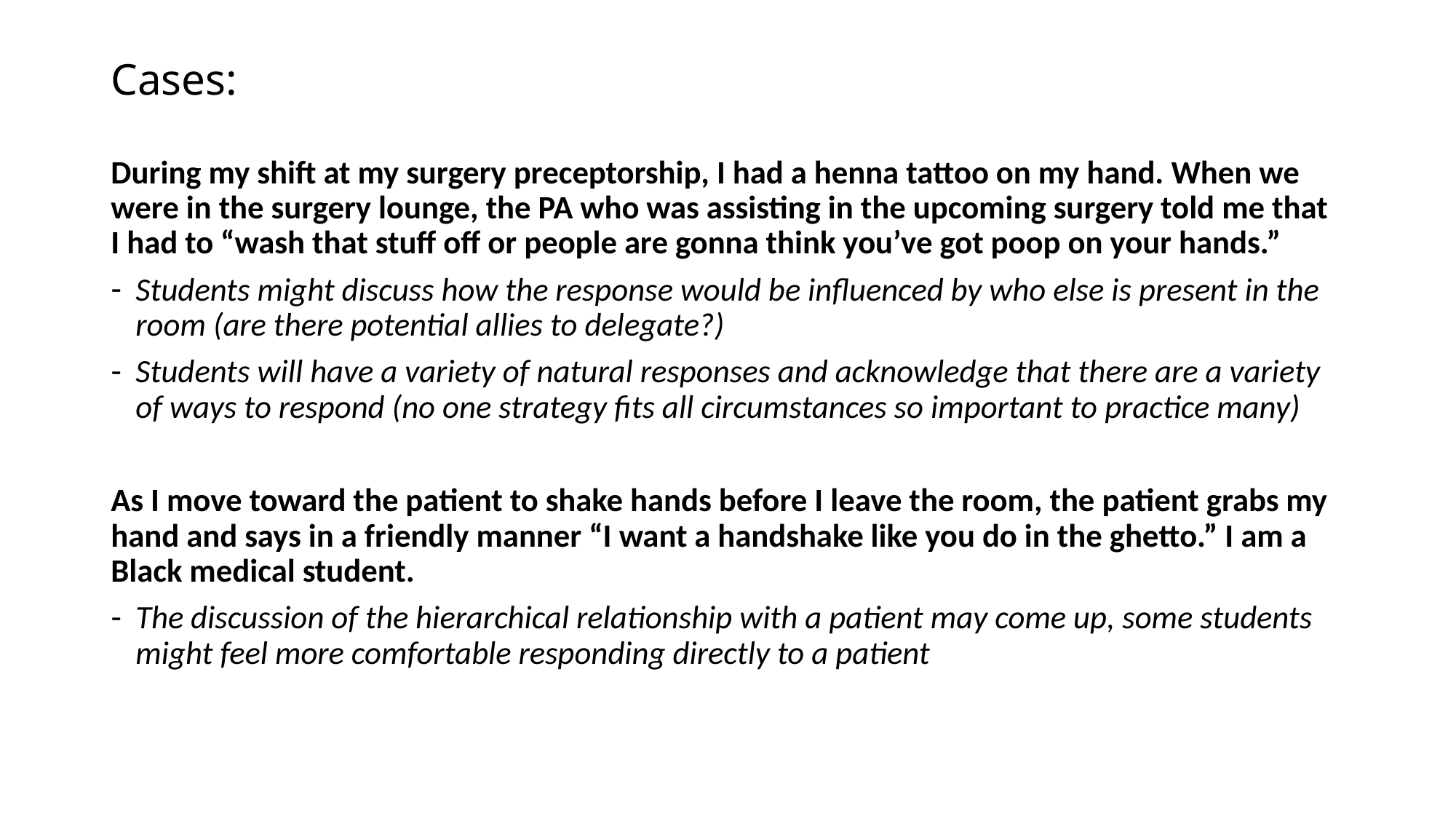

# Cases:
During my shift at my surgery preceptorship, I had a henna tattoo on my hand. When we were in the surgery lounge, the PA who was assisting in the upcoming surgery told me that I had to “wash that stuff off or people are gonna think you’ve got poop on your hands.”
Students might discuss how the response would be influenced by who else is present in the room (are there potential allies to delegate?)
Students will have a variety of natural responses and acknowledge that there are a variety of ways to respond (no one strategy fits all circumstances so important to practice many)
As I move toward the patient to shake hands before I leave the room, the patient grabs my hand and says in a friendly manner “I want a handshake like you do in the ghetto.” I am a Black medical student.
The discussion of the hierarchical relationship with a patient may come up, some students might feel more comfortable responding directly to a patient

## Slide 18
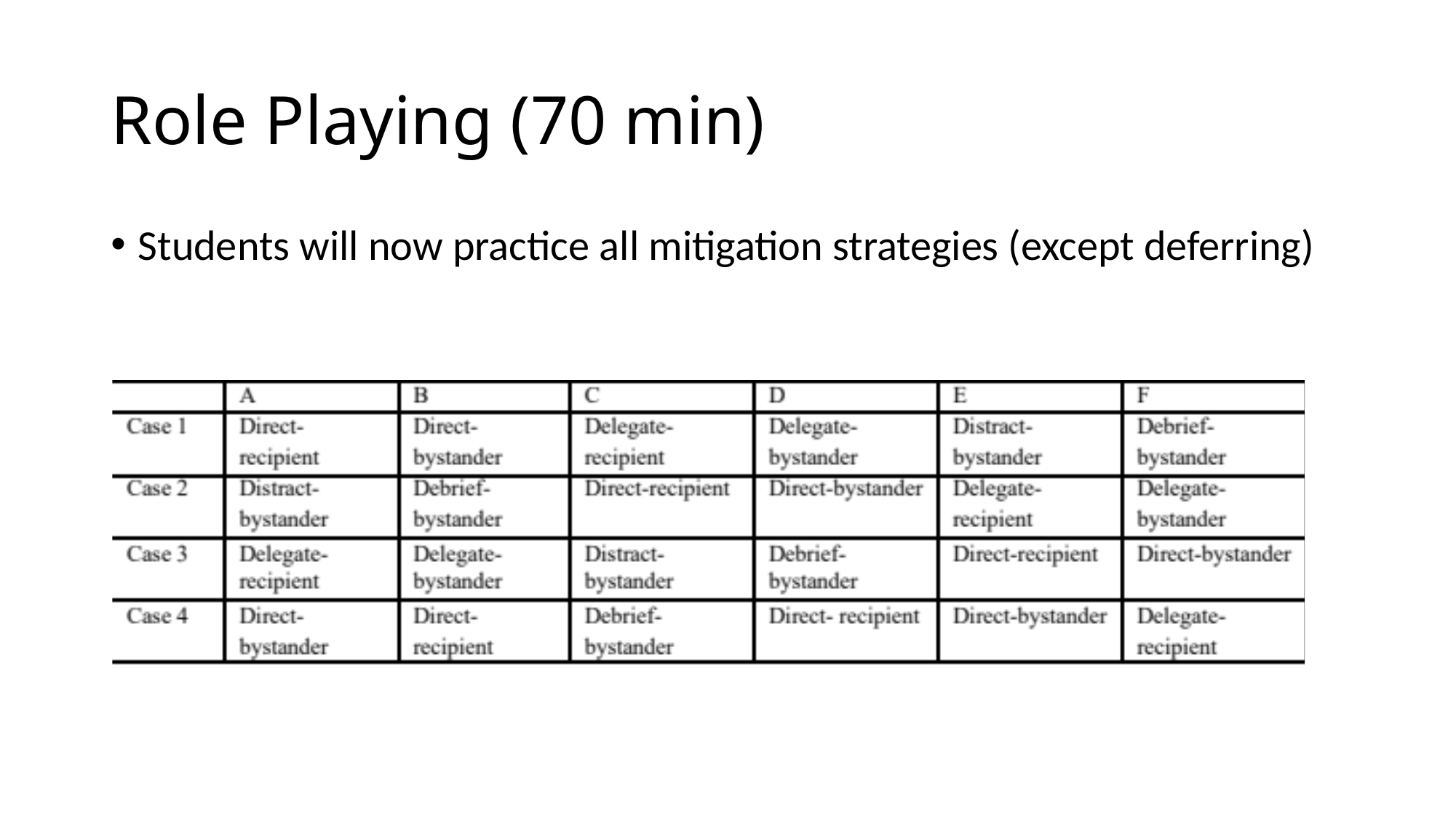

# Role Playing (70 min)
Students will now practice all mitigation strategies (except deferring)

## Slide 19
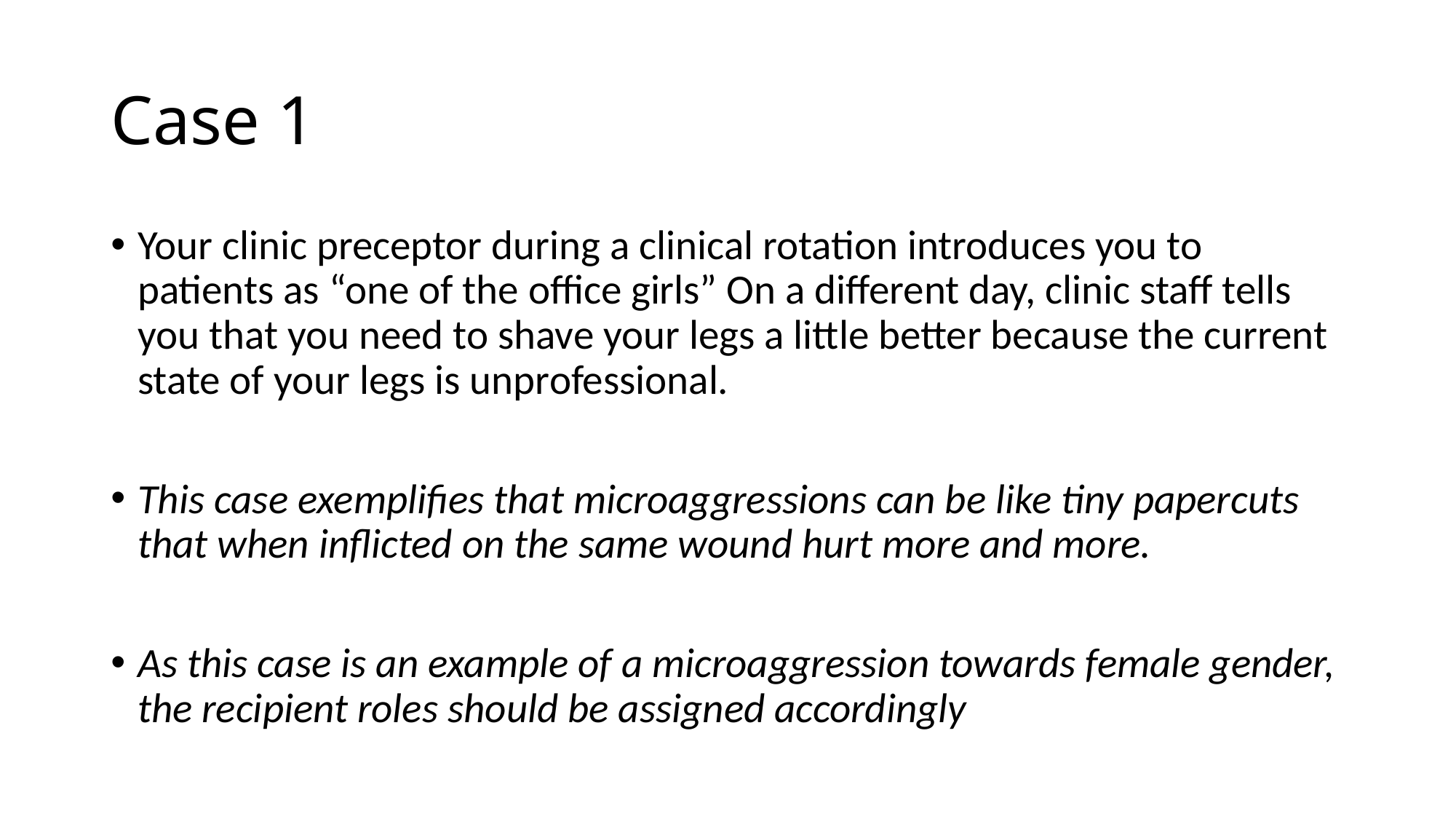

# Case 1
Your clinic preceptor during a clinical rotation introduces you to patients as “one of the office girls” On a different day, clinic staff tells you that you need to shave your legs a little better because the current state of your legs is unprofessional.
This case exemplifies that microaggressions can be like tiny papercuts that when inflicted on the same wound hurt more and more.
As this case is an example of a microaggression towards female gender, the recipient roles should be assigned accordingly

## Slide 20
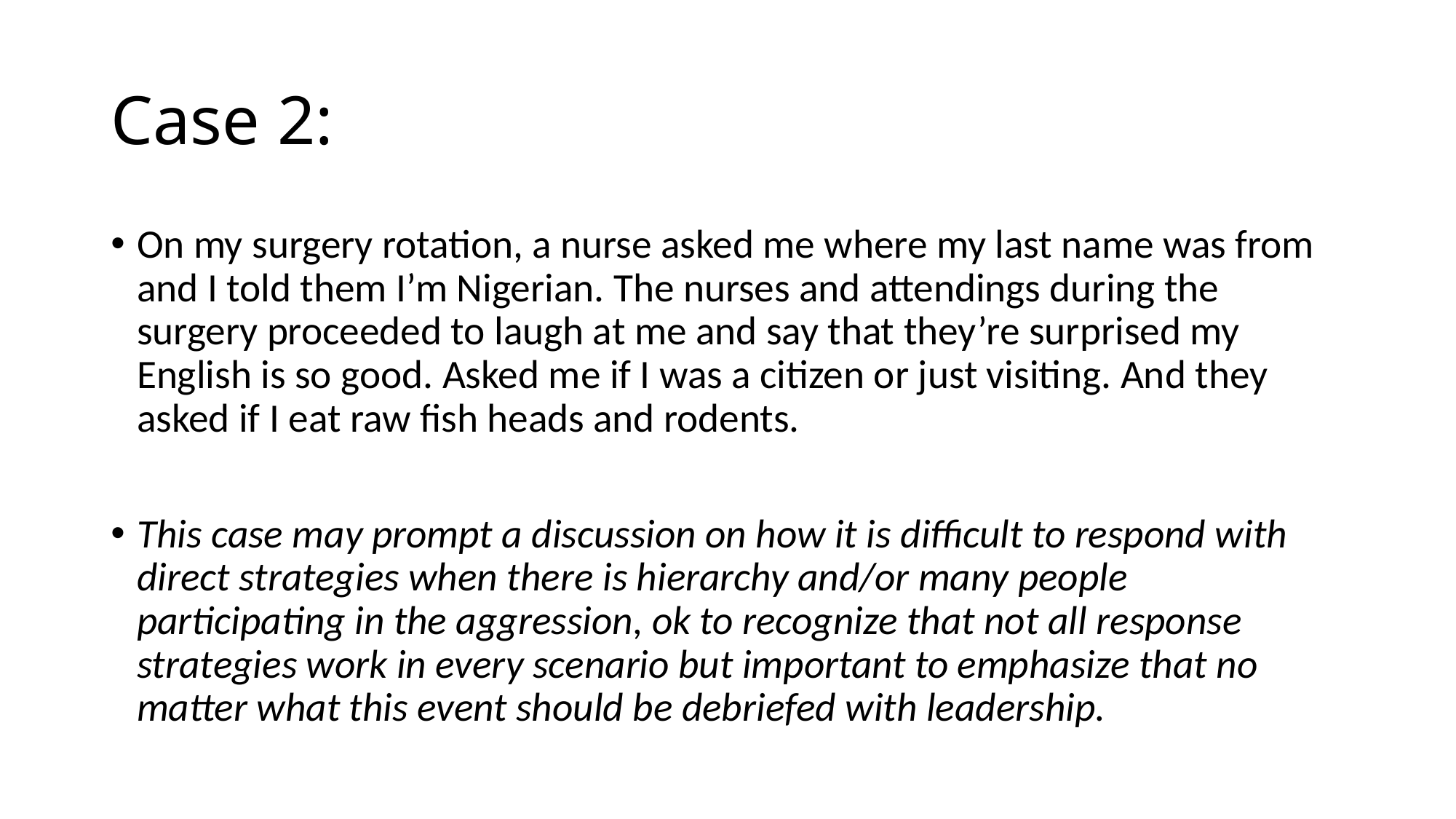

# Case 2:
On my surgery rotation, a nurse asked me where my last name was from and I told them I’m Nigerian. The nurses and attendings during the surgery proceeded to laugh at me and say that they’re surprised my English is so good. Asked me if I was a citizen or just visiting. And they asked if I eat raw fish heads and rodents.
This case may prompt a discussion on how it is difficult to respond with direct strategies when there is hierarchy and/or many people participating in the aggression, ok to recognize that not all response strategies work in every scenario but important to emphasize that no matter what this event should be debriefed with leadership.

## Slide 21
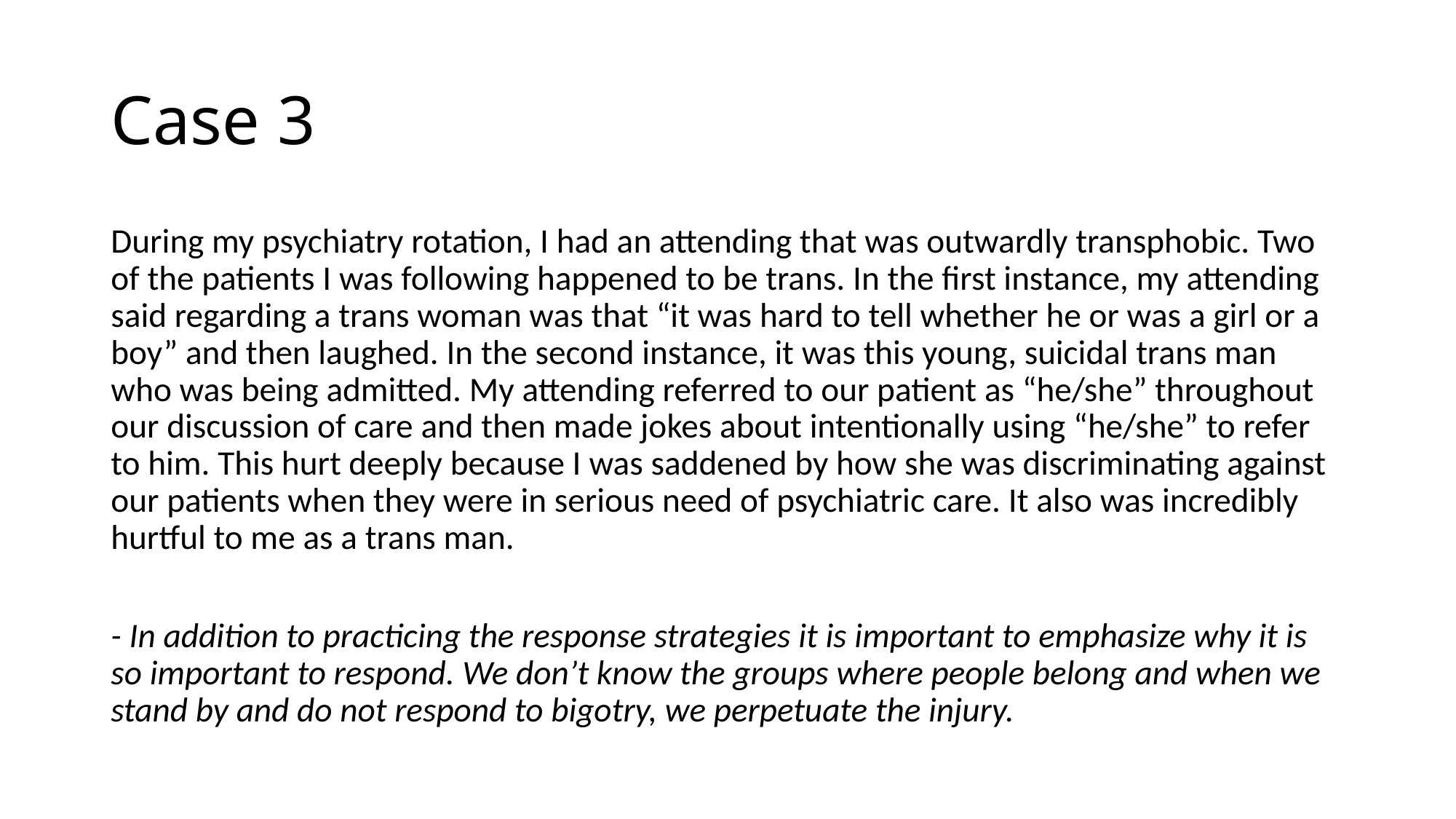

# Case 3
During my psychiatry rotation, I had an attending that was outwardly transphobic. Two of the patients I was following happened to be trans. In the first instance, my attending said regarding a trans woman was that “it was hard to tell whether he or was a girl or a boy” and then laughed. In the second instance, it was this young, suicidal trans man who was being admitted. My attending referred to our patient as “he/she” throughout our discussion of care and then made jokes about intentionally using “he/she” to refer to him. This hurt deeply because I was saddened by how she was discriminating against our patients when they were in serious need of psychiatric care. It also was incredibly hurtful to me as a trans man.
- In addition to practicing the response strategies it is important to emphasize why it is so important to respond. We don’t know the groups where people belong and when we stand by and do not respond to bigotry, we perpetuate the injury.

## Slide 22
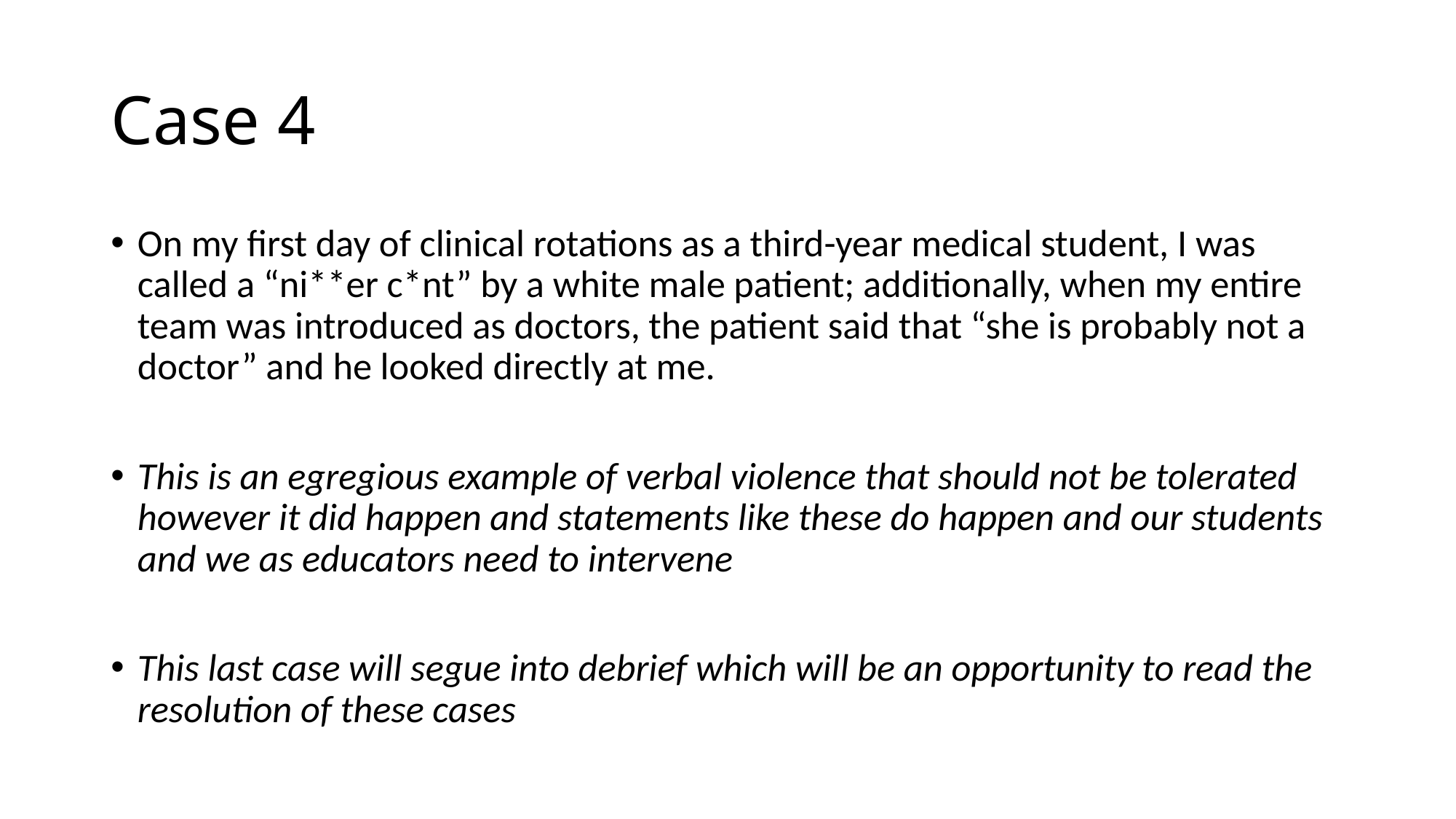

# Case 4
On my first day of clinical rotations as a third-year medical student, I was called a “ni**er c*nt” by a white male patient; additionally, when my entire team was introduced as doctors, the patient said that “she is probably not a doctor” and he looked directly at me.
This is an egregious example of verbal violence that should not be tolerated however it did happen and statements like these do happen and our students and we as educators need to intervene
This last case will segue into debrief which will be an opportunity to read the resolution of these cases

## Slide 23
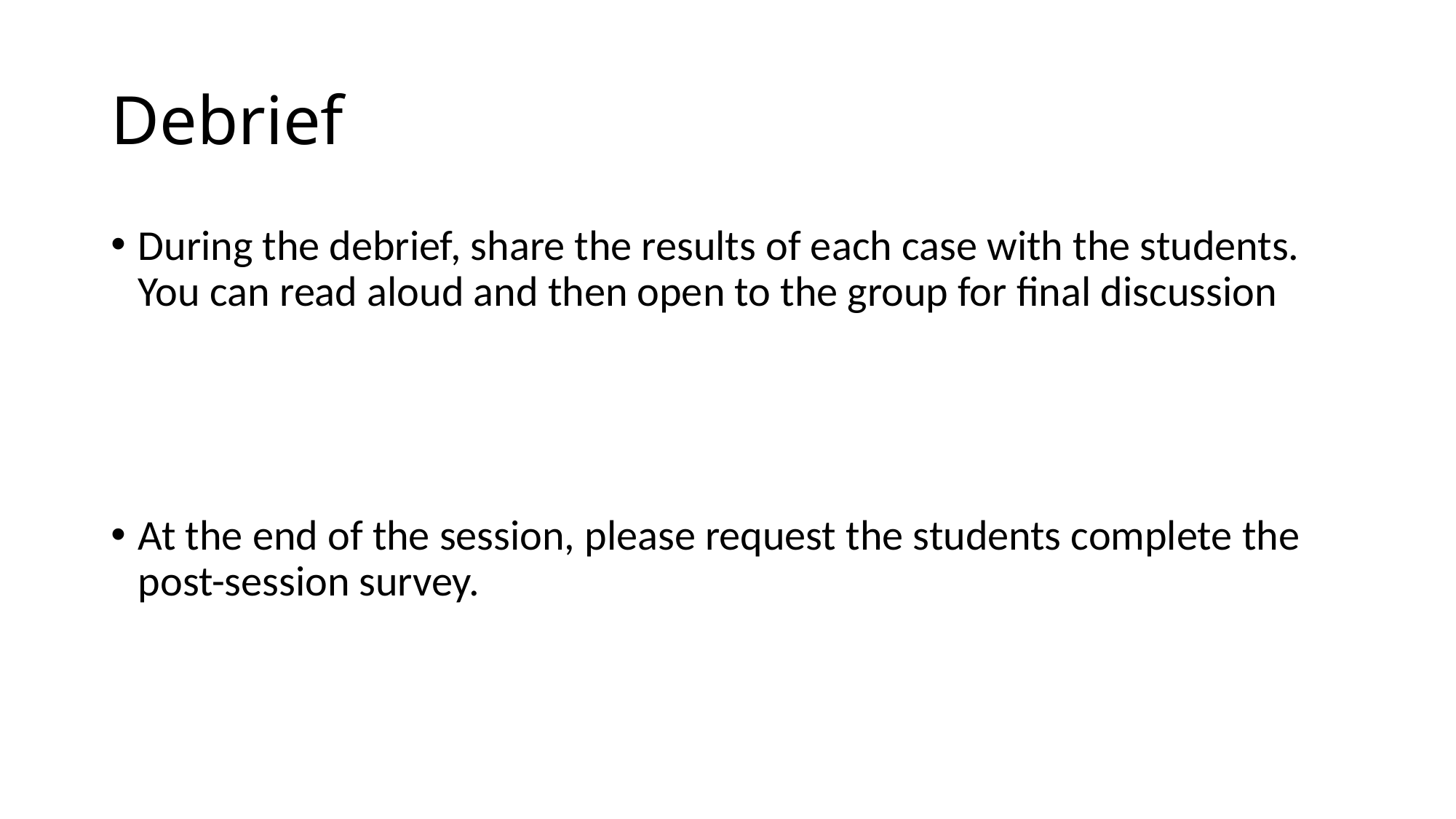

# Debrief
During the debrief, share the results of each case with the students. You can read aloud and then open to the group for final discussion
At the end of the session, please request the students complete the post-session survey.

## Slide 24
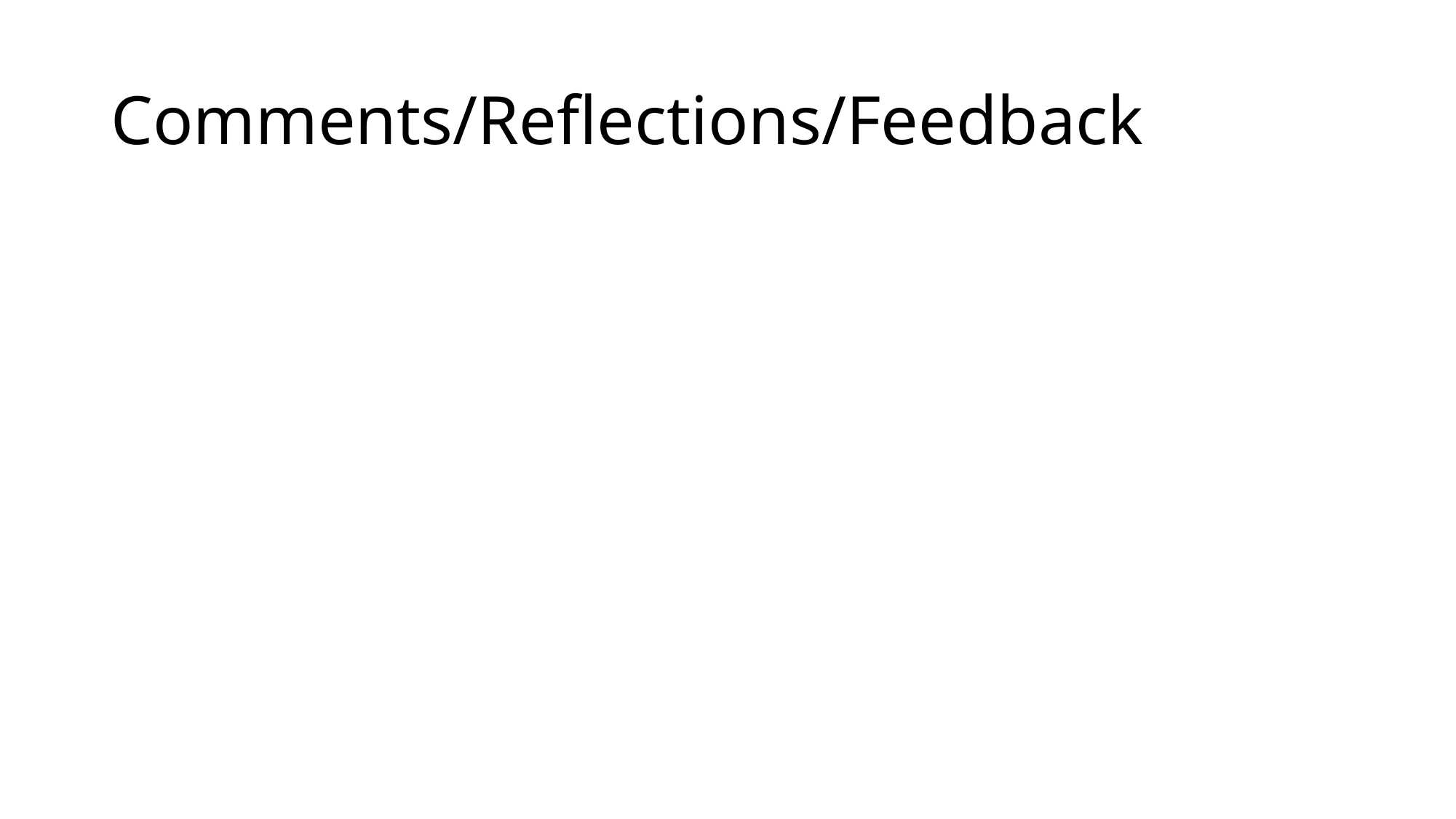

# Comments/Reflections/Feedback
