# Supplementary material for: What Happened and Why: Responding to Racism, Discrimination, and Microaggressions in the Clinical Learning Environment
Source: MedEdPORTAL. 2022 Nov 1;18:11280. doi: 10.15766/mep_2374-8265.11280 (PMC9622434; doi:10.15766/mep_2374-8265.11280)
Supplement: Supplementary file 1 — Facilitator Guide.docxStudent Guide.docxRDM Faculty Development.pptxGuide for Implementation.docxPreworkshop Survey.docxPostworkshop Survey.docx [file mep_2374-8265.11280-s001.zip › D. Guide for Implementation.docx]

## **Appendix D: Guide for Faculty Implementation:**

**Ongoing, but start at least 6 months prior to the session—begin collecting real student cases**

- At our institution, we used “informal” methods for soliciting these stories. Specifically, we reached out to classmates over social media (class Facebook and/or Discord channel) to submit these stories. We also collected stories by word-of-mouth. These methods have the benefit of being accessible to students who might be concerned about reporting incidents to more formal channels (Deans’ or Student Affairs’ offices).
- We relied on peer-to-peer contact for advertising about these cases. Depending on the culture at your institution, faculty can take a more active role in this process. Ultimately, all stories shared for cases should be offered without coercion and with consent from the students involved.

**Outreach to students who submitted a case to anonymize cases and write case resolutions**

- Once again, we relied on peer-to-peer case development. One student leader on our development team was the point person for contacting students whose cases were included in our workshop. This allowed for continuity and confidentiality for the students. The student on our team worked directly with the case writers to 1) write the case 2) write the case resolution.
- For the case resolution, we wanted students to answer the following questions (included in the case resolution section of the workshop):
  - 1. Did you or anyone else say anything in response to these comments or this situation?
  - 2. Did anyone else react to the comments or situation?
  - 3. Were you required to work with this aggressor (patient, preceptor, fellow student, etc.) again?
  - 4. Did you report these comments or the situation to anyone or debrief with peers/friends/mentors?
- Another way to anonymize this process and make it more approachable for students would be an anonymous survey through a Google form or Survey Monkey. Our team felt it was helpful to have student to student contact when developing these difficult cases. However, this might not be a possibility in all situations.

**Identify relevant offices for students to report and/or seek support**

- As part of our needs assessment in developing this session, we identified the existing curricular elements and offices on our campus where students can report these types of incidents and/or seek support.
- Conducting a similar needs or resources assessment will be essential in developing recommendations for students reporting these types of incidents.
- Examples of such offices or support systems include offices of diversity, equity, and inclusion; offices of student life or student affairs; professionalism offices or committees; faculty advisors and coaches; near-peer advisors (students and residents); identity-oriented affinity groups (e.g. Student National Medical Association, Latino Medical Student Association)
- It is useful to include recommendations and typical procedures for reporting to these offices or how to best seek support (e.g. “This office replies quickly to emails. This committee has a reporting portal and will follow-up with a phone call.”)

**Update facilitator and student guide**

- Update the guides with your institution-specific details (resources and cases as appropriate).

**3 months prior to the session—recruit student and faculty facilitators:**

- Faculty facilitators: seek out skilled facilitators, ideally who have experience in the DEI space or personal experiences with upstanding. Not all faculty need to be clinicians, though this is helpful for situations in the clinical learning environment. There should be at least one faculty facilitator per group. Residents and fellows can also be recruited in this role. At our institution, we had a pool of faculty members who had facilitated similar sessions.
- Student facilitators: It is also helpful to have student facilitators for this session. Near peer mentoring and facilitating have many benefits and can create a more open environment for the students who are participating. At our institution, we recruited facilitators from our Office of Diversity & Inclusion student group leadership as well as fourth-year students enrolled in a Physician as Educator elective.
- Where possible, be intentional about facilitator pairings. While not all groups had students as co-facilitators, those who were newer faculty facilitators were often paired with students who helped in developing the session. Similarly, check in with faculty about whether they would feel comfortable facilitating independently or with a student or other faculty member.

**Facilitator development – days to 1 week prior to session:**

- Facilitator development sessions should be held in the days leading up to the session. We have included slides (Appendix C) and materials for these sessions, but they can be customized to your audience’s knowledge and experience.
- We hosted several instances of this facilitator development session to allow for all faculty and students to attend. This also allowed for questions and discussion amongst facilitators.

**Session:**

Pre-session survey

- Refer to Appendix E for our pre-session survey. We hosted this on our institution’s Qualtrics system. This survey was made available to students prior to the session within the student guide. We allowed for a few minutes at the beginning of the session for students to complete the pre-survey.

Session

- Logistically, this workshop can be conducted in person or over a videoconferencing platform. We aimed to have groups with 4-5 students and 1-2 facilitators.
- Refer to Appendix A for the faculty guide and Appendix B for the student guide.

Post-session survey

- Refer to Appendix F for our post-session survey. We hosted this on our institution’s Qualtrics system. We allowed for a few minutes at the end of the session (where time permitted) for students to complete the post-survey. We also sent this out after the session.
- Additionally, we sent all facilitators a brief survey for them to provide feedback on the sessions.
