# Supplementary material for: What Happened and Why: Responding to Racism, Discrimination, and Microaggressions in the Clinical Learning Environment
Source: MedEdPORTAL. 2022 Nov 1;18:11280. doi: 10.15766/mep_2374-8265.11280 (PMC9622434; doi:10.15766/mep_2374-8265.11280)
Supplement: Supplementary file 1 — Facilitator Guide.docxStudent Guide.docxRDM Faculty Development.pptxGuide for Implementation.docxPreworkshop Survey.docxPostworkshop Survey.docx [file mep_2374-8265.11280-s001.zip › Educational_Summary_Report_11280.pdf]

MedEdPORTAL® The AAMC Journal of Teaching and Learning Resources

Original Publication Open Access

What Happened and Why: Responding to Racism, Discrimination, and Microaggressions in the Clinical Learning Environment

Helio V. Neves da Silva*†, Lauren M. Heery†, William R. Cohen, Vikasini S. Mahalingam, Oluwatosin A. Adebiyi, MPH, Rita S. Lee, MD, Adom N. Netsanet, Eniola A. Ogundipe, Yasmine Dakhama, Mary L. Wang, M. Aaron Vrolijk, MS, MA, Mackenzie W. Garcia, MPH, Jacqueline Ward-Gaines, MD, Anna T. Neumeier, MD

*Corresponding author: heliondsilva@gmail.com
†Co-primary author

Abstract

Introduction: Within clinical learning environments, medical students are uniquely faced with power differentials that make acts of racism, discrimination, and microaggressions (RDM) challenging to address. Experiences of microaggressions and mistreatment are correlated with higher rates of positive depression screening and lower satisfaction with medical training. We developed a curriculum for medical students beginning clerkship rotations to promote the recognition of and response to RDM. **Methods:** Guided by generalized and targeted needs assessments, we created a case-based curriculum to practice communication responses to address RDM. The communication framework, a 6Ds approach, was developed through adaptation and expansion of established and previously learned communication upstander frameworks. Cases were collected through volunteer submission and revised to maintain anonymity. Faculty and senior medical students cofacilitated the small-group sessions. During the sessions, students reviewed the communication framework, explored their natural response strategies, and practiced all response strategies. **Results:** Of 196 workshop participants, 152 (78%) completed the evaluation surveys. Pre- and postsession survey cohort comparison demonstrated a significant increase in students' awareness of instances of RDM (from 34% to 46%), knowledge of communication strategies to mitigate RDM (presession $M = 3.4$, postsession $M = 4.6$, $p < .01$), and confidence to address RDM (presession $M = 3.0$, postsession $M = 4.4$, $p < .01$). **Discussion:** Students gained valuable communication skills from interactive sessions addressing RDM using empathy, reflection, and relatability. The workshop empowered students to feel prepared to enter professional teams and effectively mitigate harmful discourse.

Keywords
Case-Based Learning, Cultural Competence, Curriculum Development, Health Equity, Diversity, Equity, Inclusion, Anti-racism

Educational Objectives

By the end of this session, learners will be able to:

1. Identify racism, discrimination, and microaggressions (RDM) that medical students may experience in the clinical setting.
2. Identify preferred strategies when faced with bias-related conflict.
3. Practice mitigation strategies when witnessing RDM.
4. Discuss the implications of discriminatory and/or biased acts for themselves, peers, and patients.

Citation:
Neves da Silva HV, Heery LM, Cohen WR, et al. What happened and why: responding to racism, discrimination, and microaggressions in the clinical learning environment. *MedEdPORTAL*. 2022;18:11280.
https://doi.org/10.15766/mep_2374-8265.11280

Introduction

Racism, discrimination, and microaggressions (RDM) are pervasive issues plaguing students at medical institutions across the country. Medical students often do not have the tools or skills to address RDM, which leads to feelings of stress and inadequacy. Many medical schools, the Association of American Medical Colleges, and the American Medical Association have committed to incorporating anti-racist practices and creating more inclusive environments for women, underrepresented minorities, and members of the LGBTQ community.[1,2] To promote safe, inclusive learning environments, a curriculum dedicated to addressing racism and discrimination is needed.

Copyright © 2022 da Silva et al. This is an open-access publication distributed under the terms of the Creative Commons Attribution license.

The impacts of RDM on medical students have been extensively described. Anderson and colleagues conducted a national study of U.S. medical students demonstrating that they frequently experienced microaggressions in the clinical setting and that such experiences were positively correlated with higher rates of positive depression screenings and lower overall medical school satisfaction.[3] In that study, 99% of respondents reported identity-based microaggressions occurring at some point in medical school, with 34% experiencing these microaggressions almost daily. Sotto-Santiago, Mac, Duncan, and Smith found that when instances of RDM did occur, medical students frequently reported that they did not know what to do.[4] Equipping students with evidence-based tools to overcome these challenging experiences is paramount to creating a safe space for students, providers, patients, and hospital staff.[5,6]

Challenges to creating effective RDM curricula include facilitator discomfort in conducting sessions, resistance and shame felt among students, few strategies to guide moving from awareness into action, and lack of skills development on how to address RDM.[7-9] Proposed methods to reduce such barriers include providing facilitators with training to help navigate challenging discussions, practicing the application of specific tools to support students when faced with RDM, and implementing a formalized longitudinal curriculum that provides a framework to aid in the recognition and management of RDM.[10,11]

We designed this session to teach medical students entering the clinical learning environment how to respond to RDM. The goals of the session, inspired by the framework created by Mateo and Williams, are to "create systems to identify and address bias and discrimination, make the reduction of bias and discrimination an institutional priority, [and] ensure comprehensive curricula to reduce bias and discrimination."[12] We use real, anonymized cases of RDM towards medical students in the clinical setting involving patients, preceptors, and other care team members to achieve these goals. The cases are accompanied by a curriculum that guides discussion, reflection, and the application of tools for responding to RDM. This curated approach provides students with specific skills on how to practice and apply a framework when addressing various forms of RDM. Ultimately, this curricular intervention hopes to reduce the negative impacts of RDM within medical education by enhancing students' confidence and preparedness to address RDM.

The session innovatively builds upon similar curricula published in *MedEdPORTAL*.[4,13-15] Similar to the work of Acholonu, Cook, Roswell, and Greene,[13] we target medical students in the clinical year, although the timing of our curricular session occurs during the transition into the clinical learning environment. In addition to focusing on real cases of students' lived experiences on our campus to support reflection, empathy, and relatability, we also provide details about how these encounters were resolved. Second, our session not only explores responses to bias and microaggressions but also openly confronts the existence of racism and discrimination in the clinical learning environment. Third, our session uses previously developed response frameworks, just as York and colleagues[14] and Walker, Hodges, Perkins, Sim, and Harris[15] do. However, our learning activities within the session expand beyond group discussion by providing tools on how to apply specific frameworks through role-play and skills practice to advance students' ability to confront, process, and debrief RDM. Lastly, we have designed the session to be cofacilitated by faculty and senior students to optimize learning climate safety with near-peer instruction. Through these innovative approaches, learners explore their natural mitigation strategies and practice alternative response strategies, which are often more active responses, with the ultimate goal of learners playing active roles in promoting safer and more inclusive learning environments.[16]

Not only does this curriculum session advance the knowledge and skills of students to address RDM in the clinical environment, it also promotes a change in culture through the formal recognition that these behaviors are unacceptable. By empowering students to identify, process, and confront RDM in the clinical environment, thes workshop fosters a cultural change that is urgently needed in a medical education system that often propagates bias.

Methods

Program Development

Our approach to curriculum development followed Kern's six step approach.[17] Localized needs assessment guided by review of course evaluations identified curricular gaps and needs. Then, a team of senior medical students, through survey and informal interviews with peers, identified the most prevalent knowledge gaps as not knowing how to respond as an upstander or as a target of RDM. Students expressed frustration and confusion about how to confront these situations given the complications posed by power dynamics and the relationships that might be at stake. Involved students and faculty reviewed existing curricular content and identified goals and objectives to address the curricular gaps identified, build upon the existing curriculum, and develop educational strategies to implement and evaluate the new curriculum. We focused the educational objectives of the workshop to provide students with the opportunity, through

Copyright © 2022 da Silva et al. This is an open-access publication distributed under the terms of the Creative Commons Attribution license.

case-based discussion, to explore the implications of discriminatory and biased acts as well as with an environment in which to practice applying mitigation strategies in instances of RDM.

Response Strategies

In the development of these strategies, senior medical students collaborated with the deans and faculty of the Office of Student Affairs, Office of Diversity and Inclusion, and Office of Professional Excellence, as well as faculty who had implemented similar programming in the emergency medicine residency affiliated with the hospital system. We expanded upon an existing, previously vetted communication framework as part of upstander training piloted earlier in our curriculum.

We considered several paradigms for upstander intervention, including the University of California, Berkeley's CARE acronym (confront, alert, redirect, engage) for addressing microaggression in the workplace[18] and the five-step CPR (confronting prejudiced responses) model developed by Ashburn-Nardo, Morris, and Goodwin.[19] Ultimately, we refined our response strategy framework through the review and application of three previously applied upstander responses (Figure 1). The first of these frameworks, the 4 Ds approach for bystander intervention, originated decades ago in the field of sexual assault prevention. Experts have subsequently modified and adapted it for use in upstander training in clinical environments.[20-22] The second framework we incorporated was described by Washington, Birch, and Roberts.[23] The third framework expanded from an earlier curricular session at the University of Colorado School of Medicine. This session, titled What Happened and Why, incorporated upstander skills to respond to microaggressions experienced in the classroom environment. A facilitator guide and student guide detailed the communication response framework (Appendices A and B).

When selecting these strategies, we wanted to ensure we identified approaches that would be most appropriate to the unique context as well as the position and preference of a medical student.[24] Because no one response strategy would fit all circumstances, we chose a variety of response strategies with varying levels of perceived risk since the use of a given strategy might be influenced by the perceived impact of the response on the student's assessment by the faculty member. Though some response strategies could be perceived negatively, we posited that the safety of the learning environment (analogous to a therapeutic alliance between patient and physician) had already been breached when the act of RDM was committed. Thus, the onus should not be put on the student to maintain a positive learning environment in the moment. Rather, these strategies should empower the student to speak up if they were comfortable doing so or to take a step back and seek out the resources available to them provided by the school of medicine. The system, not the student, should be accountable to address the psychologically harmful act and to correct the structures reinforcing racism and oppression. We therefore

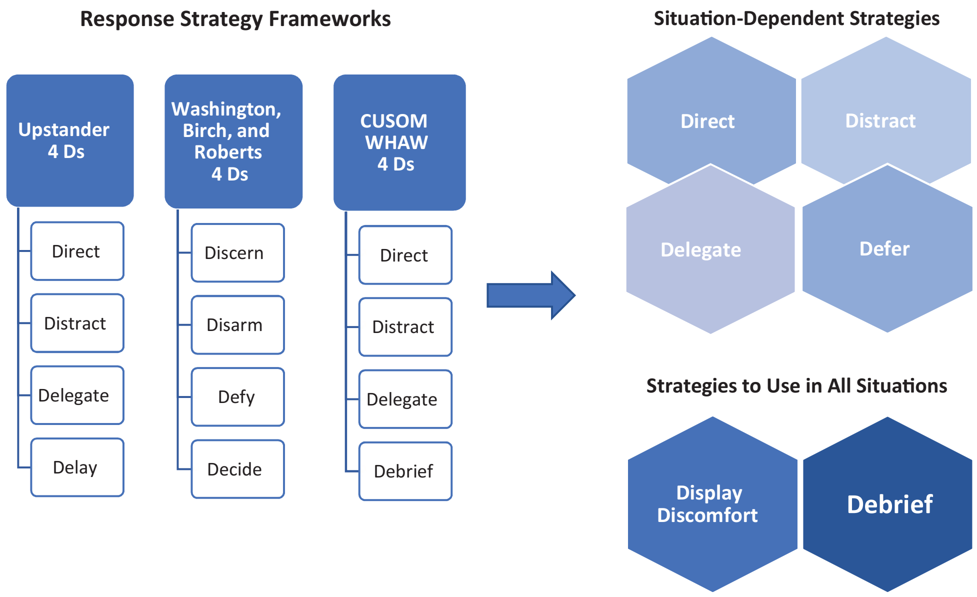

Figure 1. Situation-dependent strategies and strategies to use in all situations were adapted from previously described response strategies. Abbreviations: CUSOM, University of Colorado School of Medicine; WHAW, What Happened and Why session.

Copyright © 2022 da Silva et al. This is an open-access publication distributed under the terms of the Creative Commons Attribution license.

identified a variety of response frameworks to make them accessible to students working within the unique power dynamics of the clinical learning environment. Specifically, we wanted to highlight strategies students could use to address RDM without undermining relationships with the clinical preceptors assessing them, therapeutic relationships with patients, and collegial interactions with other care team members. For each strategy (Appendices A and B), we included examples of language to use in RDM situations with these power dynamics in mind.

Workshop Design

The student team, through social networks and personal knowledge of student experiences, obtained the voluntary submission of cases to be used in the workshop. After receiving submissions from peers, the student team and faculty mentor utilized an inductive coding technique to derive major themes from student narratives.[25] The identified prevalent topics included themes of racism, sexism, transphobia, and discrimination against patients. For each narrative selected as a teaching case, the student team contacted the submitting individual to provide additional details surrounding their response in the moment, how upstanders responded, and how the situation was resolved. After students gave consent for the use of their cases in the session, the team deidentified all cases while keeping sufficient details to maintain the spirit and truth of each incident. Just as important as the details of the event was the resolution or lack of resolution that followed. In the cases selected, the students indicated that they had reached out to a variety of existing offices, including the Office of Professionalism and Excellence, the Office of Diversity and Inclusion, and the Office of Student Life, for follow-up and resolution.

In designing the workshops, we sought to create a safe learning space given the vulnerability and confidentiality associated with these discussions. We therefore delivered the content through small-group case-based activities. The participants completed three learning activities. In the first activity, the group set ground rules and reviewed terminology and response strategy frameworks. In the second activity, students examined cases and identified their natural and immediate responses to examples of RDMs (Appendix B). In the third activity, students practiced all potential response methods through simulated interactions.

Workshop Implementation

We offered the workshop to students preparing to start their clinical clerkships. We chose to implement the workshop through cofacilitation with a student and faculty leader to model collaborative shared learning and near-peer coaching. We recruited faculty facilitators from an experienced pool of those who either taught in the preclinical upstander training session or had been trained as part of their role within the Office of Diversity and Inclusion. Student facilitators who had previously facilitated the prior upstander session, were from the student curriculum leadership team, or were part of a Physicians as Educators course were also recruited. Facilitators participated in a faculty development session (Appendix C) before leading the workshop. Due to physical distance limitations related to the COVID-19 pandemic, we delivered the session via Zoom in groups of four to six student participants with at least one faculty facilitator. These sessions were piloted as an opt-in session in May 2021 and then formally incorporated into the curriculum as a mandatory session for all students in January 2022. Additional details and the timeline for workshop implementation are available in Appendix D.

Assessment and Program Evaluation

The program evaluation was submitted to the University of Colorado Institutional Review Board and deemed exempt from formal review.

We designed a pre-/postsession survey based on the surveys from workshops developed by Acholonu, Cook, Roswell, and Greene[13] and by Sandoval and colleagues.[26] Students completed optional pre- and postsession surveys using the Qualtrics platform. We measured demographics and also allowed students to self-identify their status of underrepresented in medicine in order to intentionally include and encompass identities that might have been underrepresented in medicine but not captured by gender, sexual orientation, or race (Table 1). We measured whether participants had experienced RDM during medical school and assessed their confidence and comfort in addressing RDM using a 5-point Likert scale (1 = *strongly disagree,* 5= *strongly agree*; Appendices E and F). Individual student responses were linked by an anonymous code. For statistical analysis, through Microsoft Excel, we compared mean Likert-scale responses using *t* tests assuming unequal variance between respondent groups. We utilized paired *t* tests only with information from students who responded to both surveys using an identical unique ID number.

Results

Of the 196 participants in the workshop 152 (78%) responded to the presession survey, and 104 (53%) responded to both surveys. Ultimately, there were 77 (39%) unique ID numbers that responded to both surveys using the same ID number for both surveys. Forty-eight percent of the respondents were female, and 12% of respondents identified as underrepresented minorities in medicine.

Copyright © 2022 da Silva et al. This is an open-access publication distributed under the terms of the Creative Commons Attribution license.

Table 1. Respondents' Demographics ($N = 138$)

Self-Reported Identity Domain	No.	%
Gender identity		
Female	87	63
Male	63	46
Cisgender	136	99
Transgender	0	0
Sexual orientation		
Asexual	2	1
Bisexual	11	8
Gay	10	7
Heterosexual or straight	117	85
Lesbian	3	2
Queer	2	1
Prefer not to answer	8	6
Multiple identities	4	3
Racial and ethnic identity		
Asian	29	21
Black or African American	9	7
Hispanic or Latino/a/x/e	12	9
Native American or Alaskan Native	2	1
White	83	60
Other	10	7
Multiple identities	9	7
URM[a]		
Yes, I identify this way	59	43
No, I do not identify this way	79	57
Previously witnessed RDM in the clinical environment		
Yes	48	35
Maybe	23	17
No	67	47

Abbreviations: RDM, racism, discrimination, and microaggressions; URM, underrepresented in medicine.
[a]A specific definition of URM was not offered in the survey. Students self-reported their URM status and were subsequently able to specify their identities. This was done to include and encompass identities that might be URM but not captured by gender, sexual orientation, or race and ethnicity (e.g., asexual, deaf/hearing impaired, etc.).

One-third (34%) of respondents witnessed or thought they witnessed at least one form of microaggression or mistreatment in the clinical setting. After completion of the session, a greater proportion of students were able to identify previously witnessed or experienced microaggressions (46%, $p < .01$). Following the session, students reported increased awareness of response strategies, as measured by mean Likert score, to address instances of RDM aimed at the medical team (presession $M = 3.4$, postsession $M = 4.6$, $p < .01$), themselves (presession $M = 3.4$, postsession $M = 4.5$, $p < .01$), and patients (presession $M = 3.3$, postsession $M = 4.5$, $p < .01$).

Students additionally reported an increased level of confidence in applying communication strategies to deal with RDM in a clinical setting (presession $M = 3.1$, postsession $M = 4.4$, $p < .01$). Moreover, students reported a marked increase in their comfort addressing RDM in the clinical environment directed toward others, themselves, and patients (presession $M = 3.3$, postsession $M = 4.3$, $p < .01$; presession $M = 3.1$, postsession $M = 4.1$, $p < .01$; and presession $M = 3.4$, postsession $M = 4.3$, $p < .01$, respectively). Students reported that their comfort level in addressing RDM they may have perpetrated themselves had markedly improved (presession $M = 3.6$, postsession $M = 4.3$, $p < .01$; Figure 2).

Based on qualitative analysis, students identified the most effective components of the curriculum as being the role-play scenarios and discussion related to how different communication tools could be used for specific cases. For specific student comments on the survey, see Table 2.

Discussion

This curriculum, cofacilitated by students and faculty and delivered through small-group workshops, examines cases of RDM directed toward students to promote the recognition of RDM in a clinical setting and the application of response strategies. We built upon previously described frameworks of upstander training to provide students with skills to apply a variety of mitigation strategies.[11,27] The session incorporates variations of RDM in the clinical environment, ranging from unintentional microaggression to intentional dehumanization and verbal violence. Moreover, the curriculum advances a broader effort to confront the racism and discrimination that still impact medicine.[26,28-30]

Our curriculum provides an additional resource for anti-racism education focusing not only on the identification of RDM but also on the practice and application of a variety of response strategies. Providing variation in response strategies allows for choice in strategy application. Such variation, when considering a student's comfort level and the associated perceived risk of application, may be beneficial given the complex and dynamic power hierarchy in medicine. Another strength of this curriculum is the diversity in its clinical cases and scenarios (where perpetrators include faculty, staff, and patients). This range increases the potential for the applicability of the skills practiced across clinical rotations, specialties, and situations.

We learned valuable lessons through developing and implementing the curriculum. As noted by prior authors,[8] it is imperative to recruit from within a skilled and experienced faculty pool as well as to ensure sufficient faculty development. We recruited faculty who had previously taught similar sessions, either as part of the earlier piloted session in our curriculum or as part of their work through the Office of Diversity and Inclusion. Even with that experience, our faculty disclosed feeling nervous. Interventions we included to address this included providing faculty development both through an asynchronous recording

Copyright © 2022 da Silva et al. This is an open-access publication distributed under the terms of the Creative Commons Attribution license.

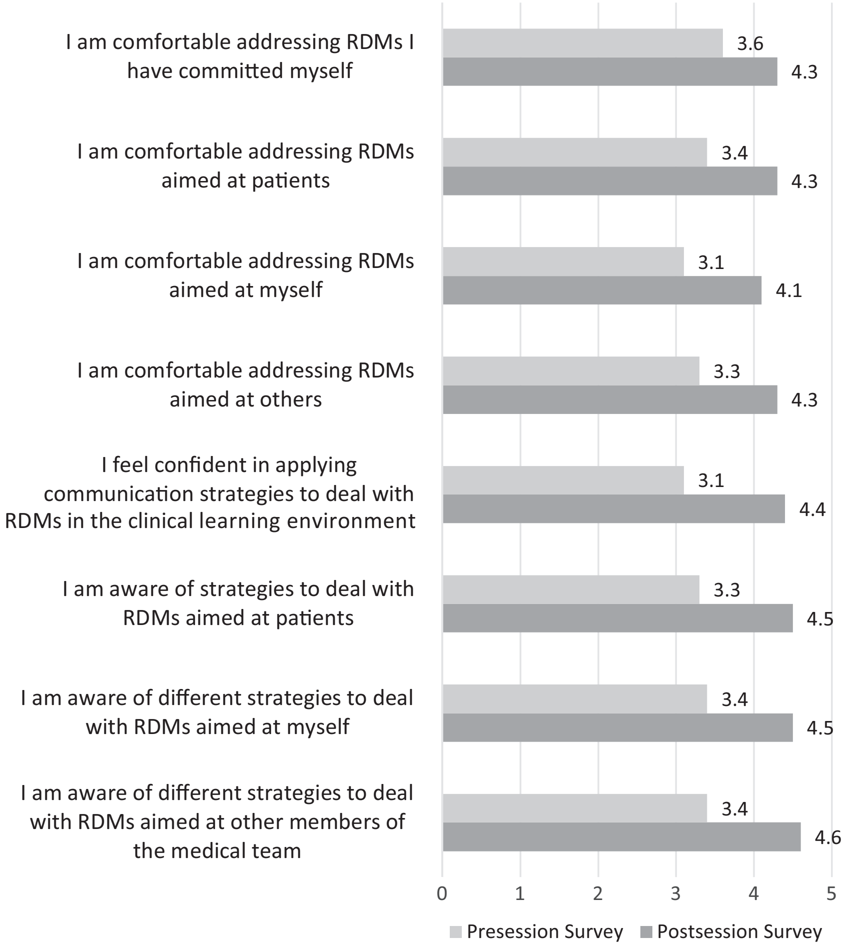

Figure 2. Comparing students' readiness to address RDMs in clinical settings using 5-point Likert-scale averages (1 = *strongly disagree,* 5 = *strongly agree*) during pre- and postsession survey analyses. For all surveyed statements, *ps* < .01. Abbreviation: RDMs, racism, discrimination, and microaggressions.

Table 2. Student Quotes

Question	Quote
What was the most beneficial part of this workshop?	"Learning how to use the different tactics in conjunction with each other—I think a combined approach is appropriate in many situations."<br>"Looking at how my own experiences affect how I would react in the situations and how to make my reactions more effective."<br>"Being able to practice in specific scenarios and think through how to deal with this situation with other people."<br>"Understanding that RDMs are still very prevalent in our system today despite efforts towards being better."
What changes could be made to make this more useful for a student of your year and training?	"Many cases were overtly offensive/hurtful. A couple more ambiguous cases would also be helpful to discuss situations that are not clearly egregious."<br>"Having multiple sessions like this during clinical year."<br>"I think it could also be helpful to have a section on how to address RDM that we may ourselves may inadvertently make."<br>"MAKE IT MANDATORY."<br>"Having common patient scenarios that 3rd year students have dealt with in the past. For example, comments that attendings make about the patient outside of the encounter."
When, during medical school, would this workshop be most useful to students?	"Every year. I feel like repeating this each year in a slightly different way is very helpful as we continue to interact in different environments and process different types of experiences."<br>"I think this is the perfect time because we don't get that many patient encounters in the first two years."<br>"I think earlier in the curriculum... before they start preceptorship or the spring of 1st year."

Abbreviation: RDM, racism, discrimination, and microaggressions.

Copyright © 2022 da Silva et al. This is an open-access publication distributed under the terms of the Creative Commons Attribution license.

of the faculty development session and through a live, just-in-time training immediately prior to the session. Additionally, cofacilitation by a student and a faculty leader helped to balance the potential burden for an individual facilitator as well as ensuring that two facilitators were focusing on the emotional environment of the session. Ultimately, although recruitment of skilled faculty is imperative, faculty development to ensure all faculty have the skills and behaviors to interrupt and address racism, bias, and microaggressions is equally important to promote cultural change. Thus, we would recommend recruiting all faculty who express interest in teaching and adapting a paired model of facilitation. Because much of the power and impact of the session derive from a review of students' lived experiences as examples, ongoing anonymous case collection is a necessary component and can be done as part of the pre- and postsession surveys.

There are limitations to this curriculum and the evaluation. Because postsession surveys were delivered immediately after the session, the evaluation represents students' knowledge and attitudes regarding confronting RDM at one measured time point. Additionally, many students either failed to respond to both surveys or did not use the same personal study ID on both surveys, preventing pairwise comparisons and the use of paired *t*-test analysis for their data. We used a per-protocol analysis and only utilized data for which there were pre- and postsession survey responses for the same study ID. This allowed pairwise comparisons and the use of paired *t* tests but did reduce the power of the analysis. Moreover, there could have been selection bias affecting the positive impact noted by the intervention as those more satisfied with the curriculum could have been more likely to complete the postsession survey. Furthermore, we did not objectively assess skills or measure whether students applied learned mitigation strategies after completion of the workshop. Given that this is a brief intervention, we cannot attest to it leading to sustained behavior change for learners. We recommend and are planning the implementation of a longitudinal anti-racist curriculum, since onetime diversity training often has limited efficacy. We agree with a recently published literature review on diversity training stating that such sessions should address the specific needs and problems of organizations, be planned in consultation with members of historically marginalized groups, and be revised and evaluated in an iterative fashion.[31,32]

Through the application of validated communication frameworks, this curriculum promotes the practice of a variety of upstander approaches to address actions of racism, discrimination, or witnessed microaggressions. Students reported improved confidence in using these mitigation strategies prior to beginning their clinical year. Longitudinal evaluation of curriculum effectiveness and impact is necessary, but the success of this session shows that it should be an integral part of inclusivity efforts in medical education.

Appendices

A. Facilitator Guide.docx
B. Student Guide.docx
C. RDM Faculty Development.pptx
D. Guide for Implementation.docx
E. Preworkshop Survey.docx
F. Postworkshop Survey.docx

All appendices are peer reviewed as integral parts of the Original Publication.

Helio V. Neves da Silva: Fourth-Year Medical Student, University of Colorado School of Medicine; ORCID: https://orcid.org/0000-0002-8572-9946

Lauren M. Heery: Fourth-Year Medical Student, University of Colorado School of Medicine

William R. Cohen: Fourth-Year Medical Student, University of Colorado School of Medicine

Vikasini S. Mahalingam: Fourth-Year Medical Student, University of Colorado School of Medicine

Oluwatosin A. Adebiyi, MPH: Fourth-Year Medical Student, University of Colorado School of Medicine

Rita S. Lee, MD: Professor, Division of General Internal Medicine, Department of Medicine, University of Colorado School of Medicine

Adom N. Netsanet: Second-Year Medical Student, University of Colorado School of Medicine

Eniola A. Ogundipe: Fourth-Year Medical Student, University of Colorado School of Medicine

Yasmine Dakhama: Fourth-Year Medical Student, University of Colorado School of Medicine

Mary L. Wang: Fourth-Year Medical Student, University of Colorado School of Medicine

M. Aaron Vrolijk, MS, MA: Fourth-Year Medical Student, University of Colorado School of Medicine

Mackenzie W. Garcia, MPH: Fourth-Year Medical Student, University of Colorado School of Medicine

Jacqueline Ward-Gaines, MD: Assistant Professor, Department of Emergency Medicine, University of Colorado School of Medicine

Copyright © 2022 da Silva et al. This is an open-access publication distributed under the terms of the Creative Commons Attribution license.

Anna T. Neumeier, MD: Assistant Professor, Division of Pulmonary Sciences & Critical Care, Department of Medicine, University of Colorado School of Medicine

Acknowledgments
This work was only possible due to activists, advocates, and scholars who have implemented training with the goals of creating safer and more inclusive environments. We want to particularly acknowledge the contribution of students and faculty in our institution who created the original What Happened and Why session as well as the first version of the upstander training for incoming medical students. Their work was a fundamental stepping stone for the creation of this project. Thank you to the following students and faculty: Ariel Kiyomi Daoud, BA, Stephanie Nwagwu, BSEd, Simon Feseha, MD, BA, Marcus Marable, BA, Carley Little, MD, MS, Deborah Seymour, PsyD, Regina Richards, PhD, and Paritosh Kaul, MD.

Disclosures
None to report.

Funding/Support
None to report.

Prior Presentations
Neves da Silva HV, Heery LM, Cohen W, et al. What happened and why: responding to racism, discrimination, and microaggressions in the clinical learning environment. Poster presented at: University of Colorado School of Medicine Academy of Medical Educators Education & Innovation Week; March 2, 2022; Aurora, CO.

Ethical Approval
The University of Colorado Institutional Review Board deemed further review of this project not necessary.

References

1. AMA Board of Trustees pledges action against racism, police brutality. American Medical Association. June 7, 2020. Accessed September 1, 2022. https://www.ama-assn.org/press-center/ama-statements/ama-board-trustees-pledges-action-against-racism-police-brutality
2. Skorton DJ. AAMC statement on police brutality and racism in America and their impact on health. Association of American Medical Colleges. June 1, 2020. Accessed September 1, 2022. https://www.aamc.org/news-insights/press-releases/aamc-statement-police-brutality-and-racism-america-and-their-impact-health
3. Anderson N, Lett E, Asabor EN, et al. The association of microaggressions with depressive symptoms and institutional satisfaction among a national cohort of medical students. *J Gen Intern Med*. 2022;37(2):298-307. https://doi.org/10.1007/s11606-021-06786-6
4. Sotto-Santiago S, Mac J, Duncan F, Smith J. “I didn’t know what to say”: responding to racism, discrimination, and microaggressions with the OWTFD approach. *MedEdPORTAL*. 2020;16:10971. https://doi.org/10.15766/mep_2374-8265.10971
5. Overland MK, Zumsteg JM, Lindo EG, et al. Microaggressions in clinical training and practice. *PM R*. 2019;11(9):1004-1012. https://doi.org/10.1002/pmrj.12229
6. Espaillat A, Panna DK, Goede DL, Gurka MJ, Novak MA, Zaidi Z. An exploratory study on microaggressions in medical school: what are they and why should we care? *Perspect Med Educ*. 2019;8(3):143-151. https://doi.org/10.1007/s40037-019-0516-3
7. Gonzalez CM, Garba RJ, Liguori A, Marantz PR, McKee MD, Lypson ML. How to make or break implicit bias instruction: implications for curriculum development. *Acad Med*. 2018;93(11S):S74-S81. https://doi.org/10.1097/ACM.0000000000002386
8. Gonzalez CM, Deno ML, Kintzer E, Marantz PR, Lypson ML, McKee MD. A qualitative study of New York medical student views on implicit bias instruction: implications for curriculum development. *J Gen Intern Med*. 2019;34(5):692-698. https://doi.org/10.1007/s11606-019-04891-1
9. Edgoose JYC, Brown Speights JS, White-Davis T, et al. Teaching about racism in medical education: a mixed-method analysis of a train-the-trainer faculty development workshop. *Fam Med*. 2021;53(1):23-31. https://doi.org/10.22454/FamMed.2021.408300
10. Ruben M, Saks NS. Addressing implicit bias in first-year medical students: a longitudinal, multidisciplinary training program. *Med Sci Educ*. 2020;30(4):1419-1426. https://doi.org/10.1007/s40670-020-01047-3
11. Sukhera J, Watling C. A framework for integrating implicit bias recognition into health professions education. *Acad Med*. 2018;93(1):35-40. https://doi.org/10.1097/ACM.0000000000001819
12. Mateo CM, Williams DR. More than words: a vision to address bias and reduce discrimination in the health professions learning environment. *Acad Med*. 2020;95(12S):S169-S177. https://doi.org/10.1097/ACM.0000000000003684
13. Acholonu RG, Cook TE, Roswell RO, Greene RE. Interrupting microaggressions in health care settings: a guide for teaching medical students. *MedEdPORTAL*. 2020;16:10969. https://doi.org/10.15766/mep_2374-8265.10969
14. York M, Langford K, Davidson M, et al. Becoming active bystanders and advocates: teaching medical students to respond to bias in the clinical setting. *MedEdPORTAL*. 2021;17:11175. https://doi.org/10.15766/mep_2374-8265.11175
15. Walker VP, Hodges L, Perkins M, Sim M, Harris C. Taking the VITALS to interrupt microaggressions. *MedEdPORTAL*. 2022;18:11202. https://doi.org/10.15766/mep_2374-8265.11202

Copyright © 2022 da Silva et al. This is an open-access publication distributed under the terms of the Creative Commons Attribution license.

16. Gold MA, Rosenthal SL, Wainberg ML. Walking on eggshells with trainees in the clinical learning environment—avoiding the eggshells is not the answer. *JAMA Pediatr*. 2019;173(10):907-908. https://doi.org/10.1001/jamapediatrics.2019.2501

17. Thomas PA, Kern DE, Hughes MT, Chen BY, eds. *Curriculum Development for Medical Education: A Six-Step Approach*. 3rd ed. Johns Hopkins University Press; 2016.

18. Berkeley Center for Support and Intervention. Bears That CARE. UC Berkeley. Accessed 2022. https://csi.berkeley.edu/prevention/btc/

19. Ashburn-Nardo L, Morris KA, Goodwin SA. The confronting prejudiced responses (CPR) model: applying CPR in organizations. *Acad Manage Learn Educ.* 2008;7(3):332-342. https://doi.org/10.5465/amle.2008.34251671

20. An overview of the Green Dot strategy. California Coalition Against Sexual Assault. Accessed September 1, 2022. http://www.calcasa.org/wp-content/uploads/2011/04/Curriculum-Summary.pdf

21. Emory Department of Medicine. Medicine grand rounds: from bystander to upstander: advocacy through action 2/04/20. YouTube. February 11, 2020. Accessed September 1, 2022. https://www.youtube.com/watch?v=YsfvDAeMqdI

22. McMahon S, Banyard VL. When can I help? A conceptual framework for the prevention of sexual violence through bystander intervention. *Trauma Violence Abuse*. 2012;13(1):3-14. https://doi.org/10.1177/1524838011426015

23. Washington EF, Birch AH, Roberts LM. When and how to respond to microaggressions. Harvard Business Review. July 3, 2020. Accessed September 1, 2022. https://hbr.org/2020/07/when-and-how-to-respond-to-microaggressions

24. Bullock JL, O'Brien MT, Minhas PK, Fernandez A, Lupton KL, Hauer KE. No one size fits all: a qualitative study of clerkship medical students' perceptions of ideal supervisor responses to microaggressions. *Acad Med*. 2021;96(11S):S71-S80. https://doi.org/10.1097/ACM.0000000000004288

25. Cristancho SM, Goldszmidt M, Lingard L, Watling C. Qualitative research essentials for medical education. *Singapore Med J*. 2018;59(12):622-627. https://doi.org/10.11622/smedj.2018093

26. Sandoval RS, Afolabi T, Said J, Dunleavy S, Chatterjee A, Ölveczky D. Building a tool kit for medical and dental students: addressing microaggressions and discrimination on the wards. *MedEdPORTAL*. 2020;16:10893. https://doi.org/10.15766/mep_2374-8265.10893

27. Tenney L. Being an active bystander: strategies for challenging the emergence of bias. The Ohio State University Kirwan Institute for the Study of Race and Ethnicity. Accessed September 1, 2022. https://kirwaninstitute.osu.edu/sites/default/files/2019-08/Active_Bystander_Handout_2019.pdf

28. Vyas DA, Eisenstein LG, Jones DS. Hidden in plain sight—reconsidering the use of race correction in clinical algorithms. *N Engl J Med*. 2020;383(9):874-882. https://doi.org/10.1056/NEJMms2004740

29. Ackerman-Barger K, Jacobs NN, Orozco R, London M. Addressing microaggressions in academic health: a workshop for inclusive excellence. *MedEdPORTAL*. 2021;17:11103. https://doi.org/10.15766/mep_2374-8265.11103

30. Amutah C, Greenidge K, Mante A, et al. Misrepresenting race—the role of medical schools in propagating physician bias. *N Engl J Med*. 2021;384(9):872-878. https://doi.org/10.1056/NEJMms2025768

31. Devine PG, Ash TL. Diversity training goals, limitations, and promise: a review of the multidisciplinary literature. *Annu Rev Psychol*. 2022;73:403-429. https://doi.org/10.1146/annurev-psych-060221-122215

32. Chang EH, Milkman KL, Gromet DM, et al. The mixed effects of online diversity training. *Proc Natl Acad Sci U S A*. 2019;116(16):7778-7783. https://doi.org/10.1073/pnas.1816076116

Received: March 28, 2022
Accepted: August 18, 2022
Published: November 1, 2022

Copyright © 2022 da Silva et al. This is an open-access publication distributed under the terms of the Creative Commons Attribution license.
